# Tyrphostin AG490 reduces inflammation and fibrosis in neonatal obstructive nephropathy

**DOI:** 10.1371/journal.pone.0226675

**Published:** 2019-12-17

**Authors:** Mojca Gasparitsch, Alexandra Schieber, Teresa Schaubeck, Ursula Keller, Marco Cattaruzza, Bärbel Lange-Sperandio

**Affiliations:** 1 Dr. v. Hauner Children’s Hospital, Division of Pediatric Nephrology, Ludwig-Maximilians-University, Munich, Germany; 2 Department of Physiology, Ruprecht-Karls-University, Heidelberg, Germany; Anatomy, SWITZERLAND

## Abstract

**Background:**

Congenital obstructive nephropathy is the main cause of end-stage renal disease in infants and children. Renal insufficiency is due to impaired growth and maturation in the developing kidney with obstruction. Congenital obstructive nephropathy leads to cytokine mediated inflammation and the development of interstitial fibrosis. The Janus kinase-2 (JAK-2) and Signal Transducer and Activator of Transcription’-3 (STAT3) are involved in cytokine production, inflammation, and interstitial fibrosis.

**Methods:**

We studied the role of JAK2/STAT3 in a model of congenital obstructive nephropathy using unilateral ureteral obstruction (UUO) in neonatal mice at the second day of life. Cytokine production, inflammation, and interstitial fibrosis were analyzed in obstructed and sham operated kidneys of neonatal mice treated with or without JAK2/STAT3 inhibitor Tyrphostin AG490. To mimic obstruction and distension, proximal tubular cells were stretched *in vitro*.

**Results:**

We show that STAT3 is highly activated in the developing kidney with obstruction and in proximal tubular cells following stretch. JAK2/STAT3 activation mediates cytokine release and leukocyte recruitment into neonatal kidneys after UUO. Pharmacological blockade of JAK2/STAT3 by Tyrphostin AG490 reduced inflammation, tubular apoptosis, and interstitial fibrosis. JAK2/STAT3 blockade decreased pro-inflammatory and profibrotic mediators in tubular cells.

**Conclusion:**

Our findings provide evidence that JAK2/STAT3 mediates inflammation and fibrosis in the developing kidney with obstruction. Blocking JAK2/STAT3 may prove beneficial in congenital obstructive nephropathy in children.

## Introduction

Congenital obstructive nephropathy is the main cause of chronic renal failure in infants and children [1–3]. In fetal development, urinary tract obstruction impairs renal growth and development and leads to renal dysplasia. Congenital obstructive nephropathy is therefore fundamentally distinct from obstruction acquired later in life. A complex interplay of genetic and non-genetic factors regulates the development of congenital obstructive nephropathy [1–3]. Unilateral ureteral obstruction (UUO) in neonatal mice at the second day of life is a model of congenital obstructive nephropathy and studies the effects of urinary tract obstruction on renal development. Neonatal UUO reduces nephron numbers, induces interstitial fibrosis, and promotes renal insufficiency. So far, there is no treatment available to restore kidney function. Congenital obstructive nephropathies in humans develop prenatally, mostly in the midtrimester during nephrogenesis. Whereas nephrogenesis in humans finishes prenatally at 36 weeks of gestation, nephrogenesis in mice finishes postnatally 2–3 weeks after birth. Therefore, UUO in neonatal mice at the second day of life is a model for congenital obstruction and disrupts nephron development at a similar stage as observed in humans. Neonatal UUO in mice leads to renal inflammation, tubular apoptosis and interstitial fibrosis in the kidney [4, 5]. Central to these events is the cytokine-mediated influx of macrophages and T-cells into the obstructed kidney. The Janus kinase-2 (JAK-2) and Signal Transducer and Activator of Transcription-3 (STAT3) signaling pathway regulates gene expression, inflammatory reactions, and differentiation in different cells and organs [6–8]. STAT3 is an important transcription factor that is involved in cytokine-mediated signaling and development of fibrosis in a variety of organs like heart, lung, and kidney [6–8]. Numerous growth factors and cytokines, including the interferon and gp130 family (IL-6, IL-11) activate the JAK2/STAT3 signaling pathway. Upon receptor binding activation of STAT3 proceeds through phosphorylation of the janus kinase (JAK) family which in turn phosphorylates STAT3. The activated JAK2/STAT3 pathway stimulates gene expression, inflammatory reactions, and cell proliferation [6]. In the kidney, STAT3 activation increases in interstitial fibroblasts and tubular cells of the obstructed kidney in adult mice with UUO [7, 9–11]. Tyrphostin AG490 efficiently blocks the JAK2/STAT3 pathway in different models [12–18]. In the kidney, Tyrphostin AG490 protects from oxidative stress, inflammation, ischemia reperfusion injury, and sepsis [17–21]. The effect of Tyrphostin AG490 in the developing kidney with obstruction has not been addressed so far. To fill this gap, we have studied the effects of JAK2/STAT3 blockade in UUO kidneys during nephron development and could show that Tyrphostin AG490 effectively reduces inflammation and fibrosis in the neonatal kidney with obstruction.

## Materials and methods

### Experimental protocol

Two-day-old male and female WT mice (C57/BL6) were distributed into twelve groups (n = 15 in each group) receiving either subcutaneous injections of the JAK2/STAT3 tyrosine kinase inhibitor Tyrphostin AG490 (AG490) (Sigma-Aldrich, Steinheim, Germany) at 10 mg/kg BW/d dissolved in 45% DMSO with 55% NaCl (vehicle) for 1 (day 2–3 of life), 5 (day 2–7 of life) or 12 days (day 2–14 of life), or vehicle once daily [22]. Mice were subjected to complete left ureteral obstruction (UUO) or sham operation under general anesthesia with isoflurane (3–5% v/v) and oxygen at the second day of life as described before [5]. After recovery neonatal mice were returned to their mothers until sacrifice at day 3, 7 and 14 of life (1, 5 and 12 days after surgery; n = 15 per group). Tyrphostin AG490 injection volumes (7–28 μl/d) increased over time in proportion to the body weight gain in neonatal mice. Tyrphostin AG490 treated mice appeared healthy and development was unremarkable. The animal experiments were approved by the Committee for Animal Experimentation of the University of Munich (Az 55.2-1-54-2531-90-10). The kidneys were removed and either fixed in paraformaldehyde 4% for immunostaining for 24 hours or frozen in liquid nitrogen and stored at -80°C for further experiments.

### Cell culture

The PKSV-PR cell is an immortalized renal cell line derived from late proximal tubules microdissected from the kidney of a transgenic mouse carrying the SV40 large T antigen gene (Tag) placed under the control of the 5’ regulatory sequence of the L-type pyruvate kinase promoter [23, 24]. The proximal tubular cell line PKSV-PR was provided by Dr. Alain Vandewalle, INSERM, Paris. For studies involving cyclic stretch, PKSV-PR cells were grown as a monolayer on flexible collagen-coated membranes for 3 days (BioFlex Culture Plates, Flexcell, Dunn Labortechnik GmbH, Asbach, Germany) under conditions as previously described [23, 24]. The culture plates were placed on vacuum-based loading docks of the Flexercell-Strain Unit (FX-5000T) in the incubator. PKSV-PR cells were subjected to pulsatile cyclic stretch at a frequency of 1 Hz with an elongation of 20% for 2, 4, and 6 hours [25, 26]. Tyrosin kinase inhibitor Tyrphostin AG490 (Sigma-Aldrich, Steinheim, Germany) was used as JAK2/STAT3 inhibitor. Tyrphostin AG490 was added (100 **μ**M) to the medium 24 hours before cyclic stretch [27]. Controls (non-stretched cells) were exposed to identical experimental conditions but without cyclic stretch. Cells were harvested and proteins were extracted. Protein expression was analysed by Western blotting. Pro-inflammatory chemokines in supernatants were measured by ELISA.

### Identification of p-STAT3 and infiltrating leukocytes

The abundance of activated p-STAT3 and infiltrating leukocytes in the neonatal kidney were examined by immunohistochemistry. Formalin-fixed, paraffin-embedded kidney sections were subjected to antigen retrieval and incubated with either rabbit anti-mouse phospho-STAT3 antibody (Cell Signaling #9145, New England Biolab GmbH, Frankfurt, Germany) at 1:50), anti-mouse F4/80 antibody against macrophages (Santa Cruz Heidelberg, Germany, # sc-377009 at 1:50), or rat anti-human CD3 antibody (AbD Serotec # MCA1477, Duesseldorf, Germany) against T-lymphocytes at 1:50. Specificity was assessed through simultaneous staining of control sections with an unspecific, species-controlled primary antibody or preincubation of the primary antibody with blocking peptides. Biotinylated goat anti-rat IgG, rabbit anti goat IgG (Southern Biotechnology, Birmingham, USA), horse anti-mouse IgG (Cell Signaling, New England Biolab GmbH, Frankfurt, Germany), or goat anti-rabbit IgG (Santa Cruz, Heidelberg, Germany) were used as secondary antibodies. Sections were incubated with ABC reagent, detected with DAB (Vectastain, Vector Laboratories, Burlingame, CA) and counterstained with methylene blue or hematoxylin. Images were taken using the LEICA DM1000 microscope and the digital camera (LEICA ICC50HD, Wetzlar, Germany). Digital images of the sections (n = 10 in each group) were superimposed on a grid, and the number of grid points overlapping dark brown F4/80 positive macrophages was recorded for each field. Macrophages and CD3-positive lymphocytes in cortex and medulla were counted in twenty non-overlapping high-power fields at x400 magnification and were analyzed in a blinded manner. Data were expressed as the mean score ± s.e. per 20 high-power fields.

### Detection of apoptosis and proliferation

Apoptotic cells were detected by the terminal deoxynucleotidyl transferase (TdT)- mediated dUTP-biotin nick end labeling (TUNEL) assay [28]. Briefly, 4% formalin-fixed tissue sections were deparaffinized and rehydrated in ethanol, followed by incubation with proteinase K (20 **μ**g/ml). After quenching, equilibration buffer and working strength enzyme (ApopTag Peroxidase In Situ Apoptosis Detection Kit, Millipore, Billerica, Massachusetts, USA) were applied. Cells were regarded as TUNEL-positive if their nuclei were stained black and displayed typical apoptotic morphology. Apoptosis in each kidney was calculated by counting the number of TUNEL-positive tubular and interstitial cells in 20 sequentially selected fields at x400 magnification and expressed as the mean number ± s.e. per 20 high-power fields using the LEICA DM1000 microscope and the digital camera (LEICA ICC50HD, Wetzlar, Germany). For detection of proliferation formalin-fixed, paraffin-embedded kidney sections were subjected to antigen retrieval and incubated with mouse anti-rat Ki67 antibody (Dako, # M7248, Agilent Technologies, California, USA) at 1:50). Sections were incubated with ABC reagent, detected with DAB (Vectastain, Vector Laboratories, Burlingame, CA) and counterstained with hematoxylin. Digital images of the sections (n = 10 in each group) were superimposed on a grid, and the number of dark brown Ki67 positive nuclei was recorded for each field. Proliferating tubular and interstitial cells in cortex and medulla were counted in twenty non-overlapping high-power fields at x400 magnification and were analyzed in a blinded manner. Data were expressed as the mean score + s.e. per 20 high-power fields.

### Measurement of interstitial fibrosis

Interstitial collagen deposition was measured in Masson’s trichrome-stained sections. Digital images of the sections were superimposed on a grid, and the number of grid points overlapping interstitial blue-staining collagen was recorded for each field. In addition, formalin-fixed and paraffin embedded sections were subjected to antigen retrieval and incubated with goat anti-mouse collagen I antibody (dilution 1:500, Southern Biotech 1310–01, Birmingham, USA) or mouse anti-mouse α-smooth muscle actin antibody (dilution 1:5000, A2547, Sigma AldrichMO851, Steinheim, Germany). Biotinylated donkey anti goat IgG and horse anti-mouse IgG (Vector Laboratories, Burlingame, CA, USA) was used as a secondary antibody. Sections were incubated with ABC reagent, detected with DAB (Vectastain, Vector Laboratories, Burlingame, CA) and counterstained with hematoxylin. Digital images of the sections (n = 10 in each group) were superimposed on a grid, and the number of grid points overlapping collagen I fibers or α-smooth muscle actin fibers was recorded for each field. Twenty non-overlapping high-power fields at x400 magnification were analyzed in a blinded fashion using the LEICA DM1000 microscope and the digital camera (LEICA ICC50HD, Wetzlar, Germany). Data were expressed as the mean score ± s.e. per 20 high power fields.

### Western immunoblotting

Tyrphostin AG490- or vehicle-treated male and female neonatal mice underwent UUO surgery or sham operation at the second day of life for Western blot analysis. Kidneys were collected 1, 5, and 12 days after obstruction (n = 5 in each group), homogenized in protein lysis buffer (Tris 50 mM, 2% SDS, 1 mM Na_2_VO_2_) containing proteinase inhibitors (Complete Mini, Roche Diagnostics GmbH, Penzberg, Germany) and benzonase (Novagen, Merck KGaA, Darmstadt, Germany) and centrifuged for 10 minutes at 16,000 x *g*. Total lysates were used for Western blotting. In addition, cell culture experiments were performed using biomechanical stimulation. PKSV-PR tubular cells were stretched for 2, 4 and 6 hours and treated with Tyrphostin AG490 or vehicle [25, 26]. Proteins were isolated. The protein content was measured using the BCA kit (Thermo Scientific #23225, Waltham, Massachusetts, USA). Twenty micrograms of protein were separated on polyacrylamide gels at 160V for 80 minutes and blotted onto PVDF-membranes (Millipore, Schwalbach, Germany) (80 mA/membrane, 90 min). After blocking for 2 hours in Tris-buffered saline with Tween-20 containing 5% nonfat dry milk and/or BSA, blots were incubated with primary antibodies 2 hours at room temperature or at 4°C overnight. Blots were washed and incubated with horseradish peroxidase-conjugated secondary antibody for 1h at room temperature. Immune complexes were detected using enhanced chemiluminescence. Blots were exposed to X-ray films (Kodak, Stuttgart, Germany), and protein bands were quantified using densitometry. Each band represents one single mouse kidney. Rabbit anti-STAT3 antibody (#9132, 1:1500), p-STAT3 (#9145, 1:1500), rabbit anti-cleaved caspase 8 antibody (#9496, 1:500), and rabbit anti cyclin D1 (#2978, 1:1000) were purchased from Cell Signaling (Cell Signaling, New England Biolab GmbH, Frankfurt, Germany), goat anti-CCL2 (#AF479-NA, 1:1000) from R&D Systems GmbH, Wiesbaden-Nordenstadt, Germany. Mouse anti-α-smooth muscle actin antibody (1:1000) was purchased from Sigma-Aldrich, Steinheim, Germany. Mouse anti-cyclin B1 antibody (#sc245, 1:200) and mouse anti-MMP-2 (#sc10736, 1:1000) were obtained from Santa Cruz, Heidelberg, Germany.

### ELISA

Chemokines were measured in the supernatant of stretched PKSV-PR cells using ELISA for CCL5 (RANTES) (#MMR00; R&D Systems GmbH, Wiesbaden-Nordenstadt, Germany). Cell supernatants of Tyrphostin AG490 or vehicle-treated PKSV-PR cells were analyzed according to the kit user manuals. Values are means **±** s.e. of at least 5 independent experiments.

### Statistical analysis

Data are presented as mean ± SEM. Comparisons between groups were made using one-way analysis of variance followed by the Student-Newman-Keuls test. Unpaired Student’s t test was used to compare the means of two groups. Statistical significance was defined as p≤0.05. For statistical analysis SigmaStat 4.0 was used (Systat Software GmbH).

## Results

### Kidneys from neonatal UUO mice showed tubular dilatation and activated STAT3-expression

**Fig 1:**
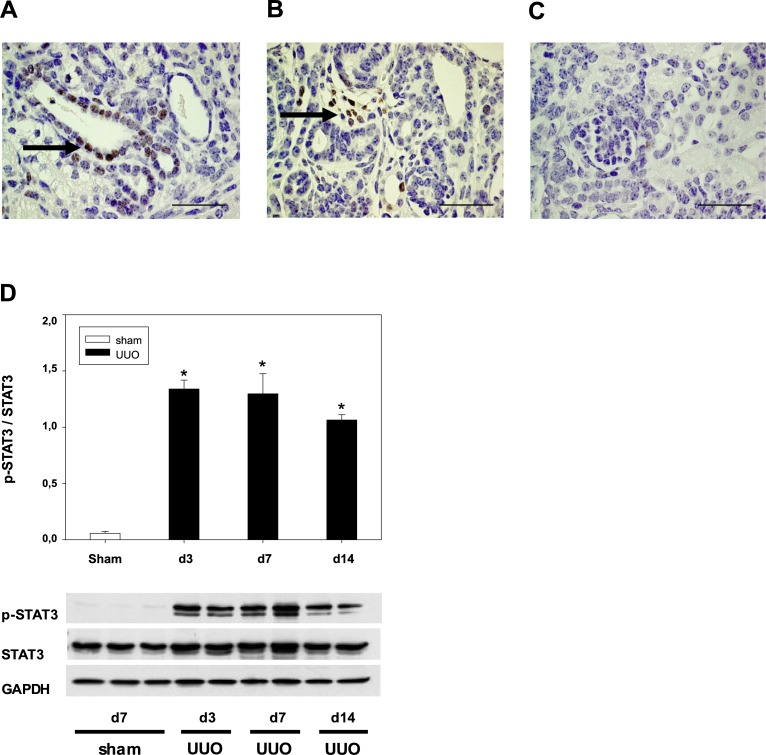
Expression of phosphorylated signal transducer and activator of transcription 3 (p-STAT3) and STAT3 following unilateral ureteral obstruction (UUO) in neonatal mice. The left ureter was ligated on the 2nd day of life. Sham operated kidneys (sham) served as control. At days 3, 7, and 14, the kidneys were removed and analyzed. Kidney tissue collected at day 7 shows activated p-STAT3 expression in dilated tubular cells **(a)** and in interstitial cells of the obstructed kidney **(b)**. Sham operated controls did not show pSTAT3 expression **(c)**. Bar = 100 **μ**m. Representative immunoblots show p-STAT3, STAT3, and GAPDH expression in sham-operated and UUO-kidneys **(d)**. Expression levels were quantified by densitometry and normalized with GAPDH. Values are means + s.e. of at least three independent experiments. *P <0.05.

Neonatal mice subjected to UUO on the second day of life showed tubular dilatation and a marked upregulation of p-STAT3, which localized to tubular (Fig 1A) and interstitial cells (Fig 1B) of the neonatal kidney. Sham operated controls did not express pSTAT3 (Fig 1C). Western blot analysis of neonatal kidneys confirmed these results. The expression levels of p-STAT3 increased after ureteral obstruction (Fig 1D). Sham-operated controls showed STAT3 but no phosphorylated STAT3 expression (Fig 1D).

### Activated STAT3 expression significantly decreased in neonatal UUO kidneys treated with Tyrphostin AG490

**Fig 2:**
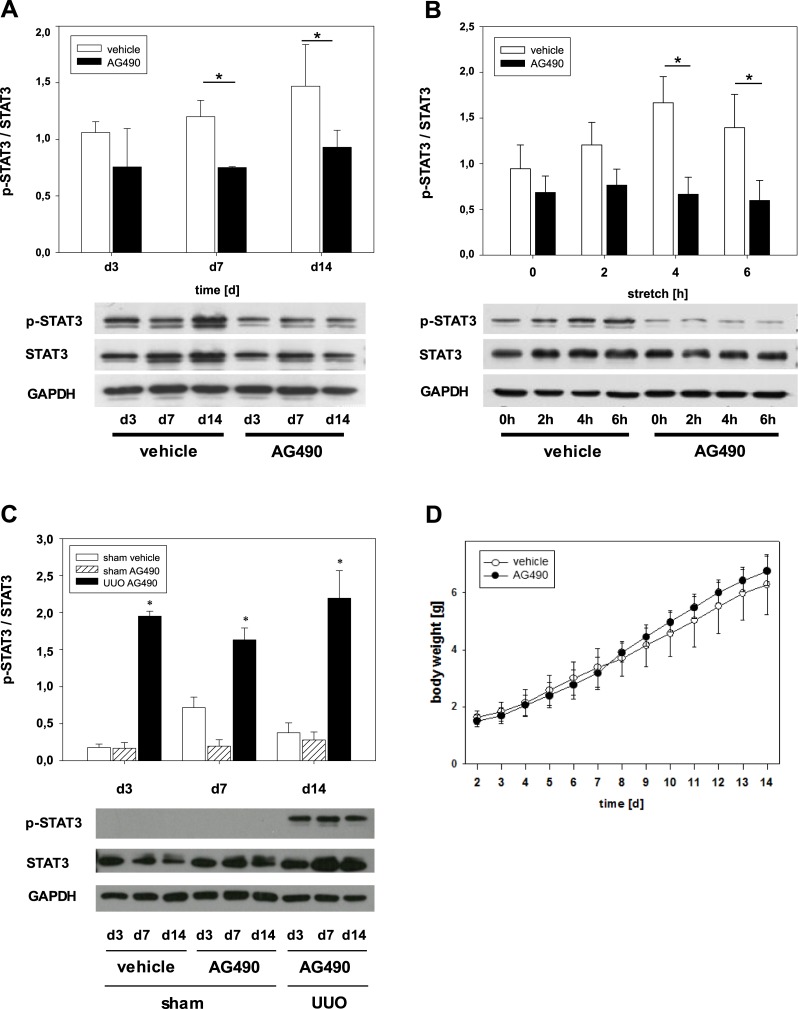
Effect of Tyrphostin AG490 on signal transducer and activator of transcription 3 (STAT3) activation. Neonatal mice were treated with Tyrphostin AG490 or vehicle and subjected to unilateral ureteral obstruction (UUO) or sham operation on the 2^nd^ day of life. At days 3, 7, and 14, kidney lysates were processed for immunoblot analysis with specific antibodies against p-STAT3, STAT3, or GAPDH. Tyrphostin AG490 decreased UUO-induced STAT3 activation. Activated STAT3 was depicted with p-STAT3/STAT3 ratio. Values are means ± s.e. of at least three independent experiments **(a)**. Tyrphostin AG490 reduced mechanical stretch-induced STAT3 activation in tubular PKSV-PR cells *in vitro* **(b)**. Tubular cells were pretreated without or with Tyrphostin AG490 for 24 hrs before treatment with cyclic stretch (20% elongation) for 2, 4, or 6 hrs. Cell lysates were analyzed for expression of p-STAT3, STAT3, and GAPDH. Activated STAT3 was depicted with p-STAT3/STAT3 ratio. Values are means + s.e. of at least three independent experiments **(b)**. Sham-operated controls showed STAT3 expression but not pSTAT3 activation **(c)**. Tyrphostin treatment showed normal body weight gain in neonatal mice **(d)**.

Treatment with the JAK2/STAT3 inhibitor Tyrphostin AG490 resulted in a significant decrease of p-STAT3 expression in obstructed kidneys compared to vehicle treated mice at day 7 and day 14 of life (Fig 2A). Next, we investigated whether biomechanical stretch of tubular PKSV-PR cells *in vitro* contributes to STAT3 activation. Cyclic stretch of tubular cells induced a marked increase of p-STAT3 expression (Fig 2B). Protein expression levels were determined by Western blotting. Tyrphostin AG490 decreased p-STAT3 activation in stretched tubular cells (Fig 2B). These findings demonstrate that Tyrphostin AG490 suppresses the activation of STAT3 *in vitro* and *in vivo*. Sham operated controls showed no pSTAT3 activation compared to UUO kidneys (Fig 2C). STAT3 expression was equal in vehicle and Tyrphostin AG490 treated mice following sham operation (Fig 2C). Daily treatment with the JAK2/STAT3 inhibitor did not induce changes in body weight gain compared to vehicle treated neonatal mice (Fig 2D).

### Tyrphostin AG490 reduced leukocyte infiltration in the neonatal UUO kidney

**Fig 3:**
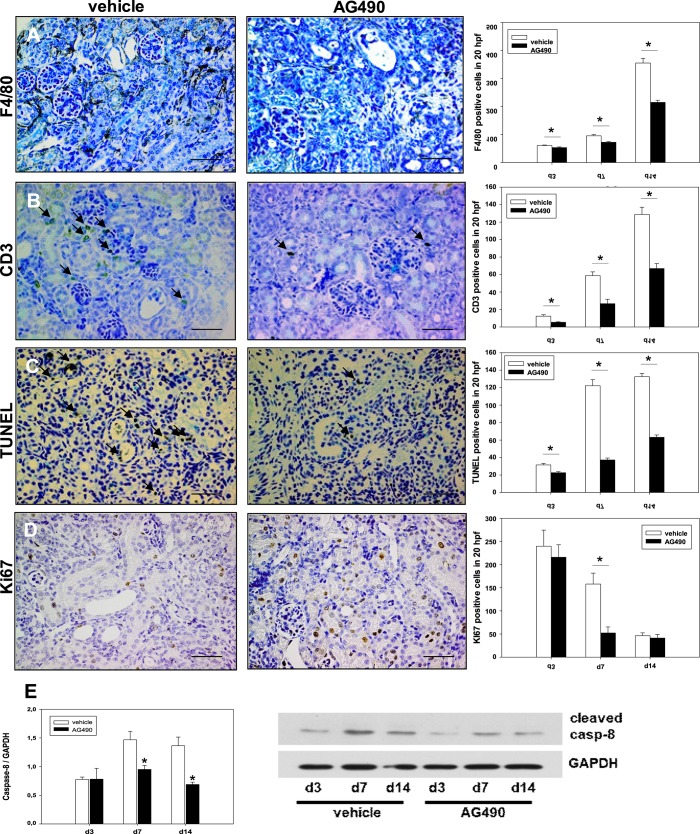
Tyrphostin AG490 reduced inflammation and tubulointerstitial injury in obstructed kidneys after unilateral ureteral obstruction (UUO) in neonatal mice. Mice received Tyrphostin AG490 or vehicle by daily injections. Renal sections of UUO- and sham-operated kidneys were stained for macrophage infiltration (F4/80 antibody) **(a)**, T-cell infiltration (CD-3 antibody) **(b)**, tubular apoptosis **(**TUNEL) **(c)**, and interstitial proliferation (Ki67 antibody) **(d)** at 3, 7, and 14 days of life and analyzed in 20 high-power fields (hpf) per section at x400. Bar = 100 **μ**m. Data are the mean ± s.e. (n = 10 in each group). Tyrphostin AG490 decreased cleaved caspase-8 expression in neonatal UUO-kidneys **(e)**. Whole kidneys were processed for western blot analysis. Each bar represents n = 3 mice. *P < 0.05.

Obstructed UUO kidneys showed a significant and progressive increase in interstitial leukocyte infiltration in vehicle and AG490 treated mice, respectively (Fig 3A and 3B). Tyrphostin AG490 reduced macrophage and T-cell infiltration in the neonatal kidney following UUO (Fig 3A and 3B). Monocytes and macrophages were stained using F4/80-antibody. The decrease of macrophages by the JAK2/STAT3 inhibitor was most pronounced at day 14 of life (Fig 3A). T-lymphocytes were examined using CD3-staining (Fig 3B). UUO induced progressive increase of T-lymphocyte infiltration in neonatal kidneys, which was significantly blocked by Tyrphostin AG490 at all time points measured (Fig 3B).

### Tyrphostin AG490 reduced tubular apoptosis in neonatal kidneys with UUO

Next, we investigated tubular apoptosis in neonatal mouse kidneys with UUO using TUNEL stain. Tubular apoptosis increased significantly in all obstructed kidneys at day 3, 7, and 14 of life (Fig 3C). Daily injections with JAK2/STAT3 inhibitor Tyrphostin AG490 reduced tubular apoptosis compared to vehicle-treated kidneys at day 3, day 7, and day 14 of life (Fig 3C). Interstitial apoptosis increased in neonatal mouse kidneys after UUO and was highest at day 14 (S1 Fig). JAK2/STAT3 blockade reduced interstitial apoptosis at all time points measured (S1 Fig). Apoptosis was also measured by caspase 8 cleavage using Western blot (Fig 3E). Cleaved caspase-8 expression increased in neonatal kidneys following ureteral obstruction. Tyrphostin AG490 significantly blocked cleaved caspase-8 expression following UUO (Fig 3E).

### Tyrphostin AG490 decreased proliferation in interstitial cells following UUO

Interstitial cells in the UUO kidneys consist of infiltrating leukocytes, fibroblasts, and myofibroblasts. Proliferation of interstitial cells was analyzed in neonatal mouse kidneys using Ki67 immunostaining (Fig 3D). Following UUO, tubular and interstitial proliferation decreased in all neonatal mouse kidneys after UUO and was lowest at day 14. Additional JAK2/STAT3 blockade further decreased interstitial proliferation reaching significance at day 7 of life (Fig 3D). By contrast, Tyrphostin AG490 increased tubular proliferation, which was significant at day 14 (S1 Fig) suggesting differential contribution of JAK2/STAT3 on interstitial and tubular proliferation following UUO.

### Tyrphostin AG490 reduced tubulointerstitial fibrosis in neonatal UUO

**Fig 4:**
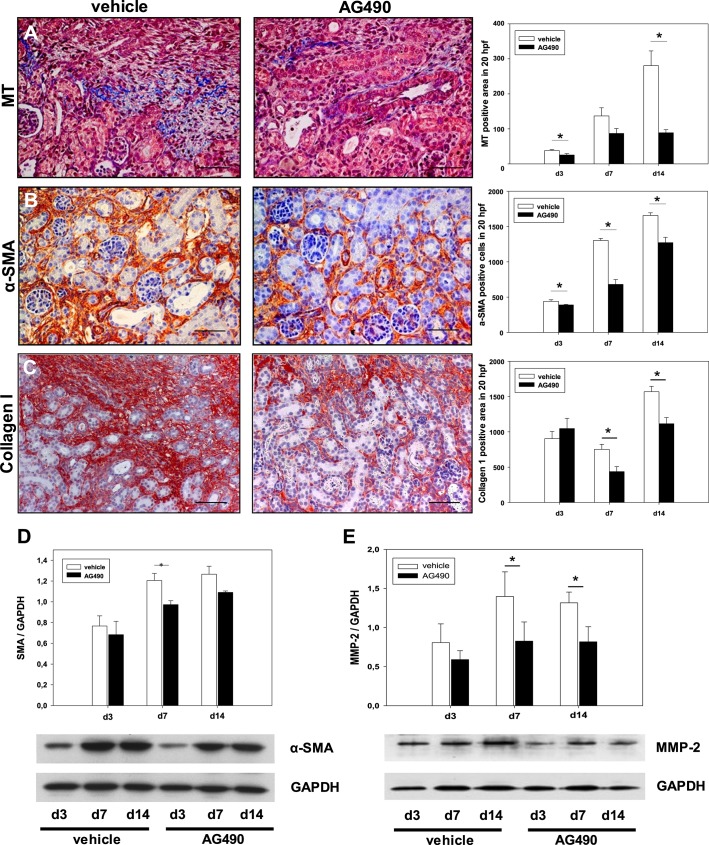
Tyrphostin AG490 reduced interstitial fibrosis in obstructed kidneys after unilateral ureteral obstruction (UUO) in neonatal mice. Renal sections of UUO- and sham-operated kidneys were stained for Masson’s Trichrome (MT) **(a)**, α-smooth muscle actin (α-SMA antibody) **(b)**, and collagen deposition (collagen I antibody) **(c)** at 3, 7, and 14 days of life and analyzed in 20 high-power fields (hpf) per section at x400. Bar = 100 μm. Data are the mean + s.e. (n = 10 in each group). Tyrphostin AG490 decreased α-SMA expression **(d)** and MMP-2 expression **(e)** in neonatal UUO-kidneys. Whole kidneys were processed for western blot analysis. Values are means + s.e. of at least three independent experiments. *P < 0.05.

To study interstitial fibrosis after UUO, Masson’s Trichrome, α-smooth muscle actin (α-SMA) and collagen I stainings of kidney sections were performed. Masson’s Trichrome, α-SMA and collagen I showed a dramatic increase of interstitial fibrosis in all obstructed kidneys (Fig 4A–4C). Daily treatment with the JAK2/STAT3 inhibitor Tyrphostin AG490 significantly reduced interstitial fibrosis, α-SMA-expression, and collagen I deposition in neonatal UUO kidneys (Fig 4A–4C). Activated myofibroblasts were also analyzed by α-SMA expression in neonatal kidneys using Western blot (Fig 4D). Following UUO, α-SMA-expression increased in the obstructed kidney, and was reduced in Tyrphostin AG490 treated mice (Fig 4D). Matrix metalloproteinases (MMP’s) are involved in the degradation of extracellular matrix. UUO induced MMP-2 expression in neonatal kidneys (Fig 4E). Tyrphostin AG490 significantly reduced MMP-2 expression in UUO-kidneys at day 7 and day 14 of life (Fig 4E).

### Tyrphostin AG490 decreased chemokine production in UUO-kidneys and stretched tubular cells

**Fig 5:**
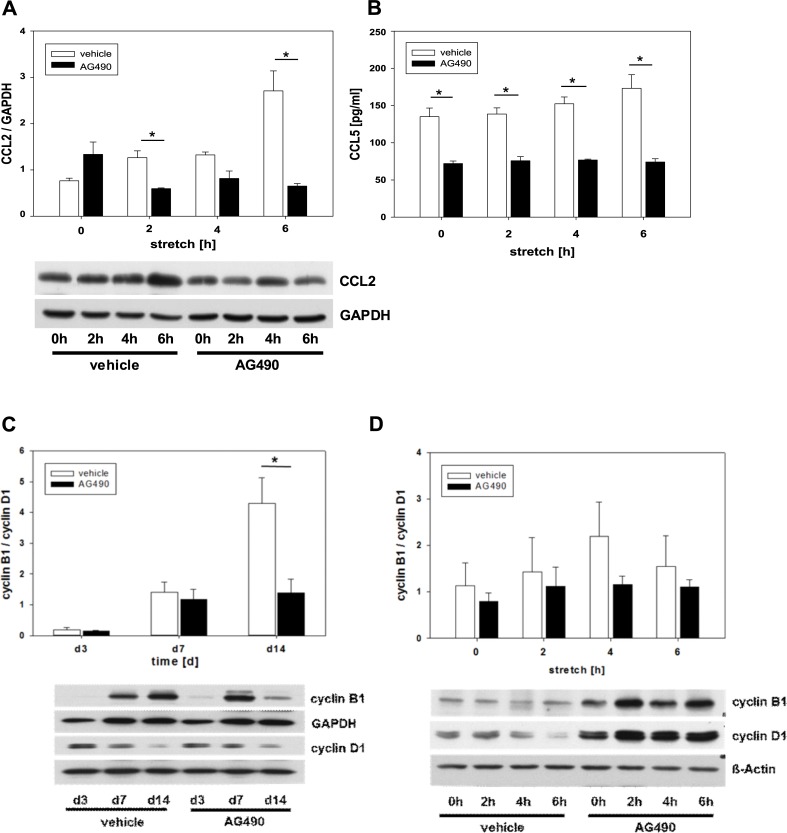
Tyrphostin AG490 reduced chemokine secretion and cell cycle arrest. Tyrphostin decreased CC-chemokine ligand 2 (CCL2) expression in stretched tubular PKSV-PR cells **(a)**. Tubular cells were grown on flexible membranes, pretreated with Tyrphostin AG490 or vehicle for 24hrs before treatment with cyclic stretch for 2, 4, or 6 hrs. Cell lysates were subjected to immunoblot analysis with specific antibodies against CCL2 or GAPDH. **(b)** Tyrphostin AG490 blocked CC-chemokine ligand 5 (CCL5) secretion. Tubular cells were stretched for 2, 4, or 6 hrs and pretreated with Tyrphostin AG490 or vehicle. Cell supernatants were analyzed for CCL5 using ELISA. Tyrphostin AG490 reduced cell cycle arrest *in vivo* **(c)** and *in vitro* **(d)**. Protein expression of cyclin B1, cyclin D1, and GAPDH was measured in obstructed kidneys **(c).** G2/M arrest is indicated by the cyclin B1/ D1 ratio. Tubular cells were stretched for 2, 4, or 6 hrs and pretreated with Tyrphostin AG490 or vehicle **(d).** Cell lysates were analyzed for expression of cyclin B1, cyclin D1, and GAPDH. Representative immunoblots are shown. Values are means + s.e. of at least three independent experiments *P <0.05.

To investigate whether Tyrphostin AG490 could inhibit the production of chemokines like CCL2 and CCL5, cultured PKSV-PR cells were exposed to cyclic stretch. Cyclic stretch stimulated CCL2-production in tubular cells (Fig 5A). Tyrphostin AG490 significantly inhibited CCL2-expression in stretched tubular cells after 2 hrs and 6 hrs of biomechanical stimulation (Fig 5A). CCL5-secretion was measured in the supernatant of stretched PKSV-PR cells using ELISA (Fig 5B). Biomechanical stretch induced CCL5 secretion by tubular cells. Tyrphostin AG490 significantly decreased CCL5-secretion in tubular PKSV-PR cells at all time points measured suggesting a contribution of the JAK2/STAT3 pathway on chemokine production and release during tubular stretch.

### G2/M-Arrest is reduced by Tyrphostin AG490 in vivo

Cell cycle arrest at G2/M in tubular cells mediates renal interstitial fibrosis [29]. To determine whether G2/M arrest was present after ureteral obstruction, the cyclin B1/ cyclin D1 ratio was measured in neonatal kidneys using Western blot (Fig 5C). Cell cycle arrest indicated by the cyclin B1/ cyclin D1 ratio increased in all obstructed kidneys when compared with sham-operated controls. Tyrphostin AG490 reduced G2/M-arrest in obstructed neonatal kidneys (Fig 5C). Biomechanical stretch of tubular PKSV-PR cells induced cell cycle arrest *in vitro* (Fig 5D). Tyrphostin AG490 reduced G2/M-arrest in PKSV-PR cells, but the difference was not significant (Fig 5D).

## Discussion

This study indicates a novel role for JAK2/STAT3 signaling in the developing kidney with obstruction. We show that JAK2/STAT3 activation mediates inflammation and fibrosis in the neonatal kidney following UUO. STAT3 expression rapidly increased after ureteral obstruction and localized to tubular and interstitial cells. The upregulation of STAT3 induced the production and secretion of chemokines and mediated leukocyte infiltration into the obstructed kidney. By using the JAK2/STAT3 inhibitor Tyrphostin AG490 chemokine secretion and leukocyte infiltration were blocked in neonatal UUO kidneys. Several studies in adult mice have shown that STAT3 blockade prevents leukocyte infiltration by downregulation of adhesion molecules (ICAM-1) and chemokines (CCL2) [9]. Our results in neonatal UUO are in line with those observations. Tyrphostin AG490 reduced macrophage and T-cell infiltration in the developing kidney with obstruction. Blocking STAT3 by Mefunidone reduced leukocyte infiltration and interstitial fibrosis in adult rats with UUO [10]. Paclitaxel reduced macrophage infiltration and fibroblast activation by inhibiting STAT3 in adult mice with UUO [11]. Similarly, pharmacological blockade and genetical knockdown of STAT3 reduced macrophage infiltration in diabetic mice and prevented glomerulopathy [7, 30]. In our study, JAK2/STAT3 blockade reduced interstitial inflammation as well as tubular apoptosis in the neonatal kidney with UUO. The response of the neonatal kidney to obstruction is clearly different from the adult kidney and shows more damage [1, 31]. Oxidative stress, hypoxia, and cytokines are responsible for tubular apoptosis following UUO. Since STAT3 regulates pro-apoptotic cytokines like TNF-α and TGF-β1, JAK2/STAT3 blockade may inhibit apoptosis by suppression of TNF-α and TGF-β1-expression in neonatal UUO-kidneys. TNF-α and TGF-β1 are able to increase STAT3-activation and amplify the apoptotic signal in UUO. We show that Tyrphostin AG490 treated mice were effectively protected from tubular and interstitial apoptosis. Accordingly, caspase 8 cleavage decreased in neonatal UUO kidneys treated with JAK2/STAT3 blockade. Similar to earlier reports, we could demonstrate better survival in tubular cells under Tyrphostin AG490 [19, 32]. Presumably, this reduction of tubular cell death in turn prevented further inflammation and reduced interstitial fibrosis. Tyrphostin AG490 increased tubular proliferation in neonatal kidneys following UUO. By contrast, proliferation of interstitial cells was blocked by Tyrphostin AG490, suggesting less proliferation of infiltrating leukocytes, fibroblasts and myofibroblasts.

Interstitial fibrosis develops in parallel with tubular injury and the inflammatory response following UUO [5]. Fibroblasts expand in the obstructed kidney due to local proliferation, TGF-β1-induced fibroblast-to-myofibroblast transition, recruitment of bone marrow-derived fibrocytes, epithelial to mesenchymal transition (EMT), macrophage to myofibroblast transition (MMT), pericyte to myofibroblast transition, and endothelial myofibroblast transition [33, 34]. Myofibroblasts are the principal source of extracellular matrix in renal fibrosis. Evaluation of myofibroblasts by α-SMA expression shows persistent activation in neonatal UUO [5]. In our study, Tyrphostin AG490 reduced α-SMA expression and extracellular matrix deposition in the neonatal kidney with obstruction. Our results are in line with recent data showing that mice with conditional deletion of STAT3 in fibroblasts have less myofibroblasts and less fibrosis [6]. Matrix metalloproteinase-2 (MMP-2) is a 72 kDa collagenase that is important in extracellular matrix metabolism and renal fibrosis. MMP-2 cleaves type IV collagen, and degrades denatured collagens. In the kidney, MMP-2 is upregulated following UUO in adult mice [35]. Reduced MMP-2 expression during UUO protects mice against renal fibrosis [35]. Since MMP-2 is a target gene of STAT3, JAK2/STAT3 blockade reduced MMP-2 expression and renal fibrosis in the developing kidney with obstruction. STAT3 seems to act as a central regulator linking tubular and interstitial cells in tissue fibrosis [6, 36]. In tubular cells STAT3 activation induces the upregulation of profibrotic factors like TGF-β1, PDGF, and TIMP-1 and controls EMT after renal injury [37–40]. In fibroblasts, STAT3-activation leads to proliferation und myofibroblast activation. In our study, Tyrphostin AG490 reduced inflammation and decreased renal fibrosis in the developing kidney with obstruction. Recently, Matsui et al. have shown that mesenchymal stem cells protect against obstruction-induced fibrosis by decreasing STAT3 activation [41]. Profibrotic STAT3 signaling critically involves the Src family of non-receptor tyrosine kinases [40, 42]. Members of the Src family kinases interact with cytosolic, nuclear and membrane proteins. They modify these proteins by phosphorylation of tyrosine residues and contribute to STAT3-dependent cellular transformation. This has been demonstrated for Fyn, a member of the Src family kinases that promotes renal fibrosis in adult mice with UUO involving STAT3 signaling [40, 42]. In contrast, genetic absence of Fyn led to antifibrotic effects and downregulation of STAT3 emphasizing the pivotal role of STAT3 in the development of fibrosis. To identify further mechanisms of STAT3 signaling, we analyzed markers of inflammation, extracellular matrix degradation, and cell cycle in stretched tubular cells *in vitro*. Mechanical stretch of tubular cells *in vitro* mimics changes in intrarenal pressure following ureteral obstruction. Mechanical stretch upregulates TGF-β1 expression and p-STAT3 in human tubular cells [43]. STAT3 inhibitor S3I-201 and knockdown by siRNA targeting human STAT3 suppress stretch-induced TGF-β1. In line with this, we could show that Tyrphostin AG490 efficiently blocked stretch-induced upregulation of p-STAT3 in tubular cells. Tyrphostin AG490 inhibited the stretch-induced expression of CCL-2 and CCL-5, thereby reducing the inflammatory response. This effect was probably due to a direct down-regulation of the expression of CCL-2 and CCL-5.

Cell cycle arrest at G2/M in renal tubular cells promotes interstitial fibrosis after UUO [5, 29]. Arrested tubular cells produce profibrogenic factors like TGF-β1 and Connective Tissue Growth Factor (CTGF). In our study, G2/M-arrest was present in neonatal UUO-kidneys and in stretched tubular cells. Tyrphostin AG490 successfully reduced G2/M-arrest in the developing kidney with obstruction, thereby leading to a sharp decline in interstitial fibrosis.

In summary, our results show that JAK2/STAT3-signaling is a key effector of disease progression in our neonatal mouse model of UUO identifying STAT3 as critical factor in mediating the inflammatory response and the development of interstitial fibrosis in the neonatal kidney with obstruction. Targeting JAK2/STAT3 may therefore offer an interesting approach for therapeutic interventions in congenital obstructive nephropathy.

## Supporting information

S1 Checklist Animal experiments were approved by the Committee for Animal Experimentation of the University of Munich and documented accordingly to the ARRIVE guidelines. (PDF)

S1 Fig **Tyrphostin AG490 reduced interstitial apoptosis (A) and increased tubular proliferation (B) in obstructed kidneys after unilateral ureteral obstruction (UUO) in neonatal mice.** Mice received Tyrphostin AG490 or vehicle by daily injections. Renal sections of UUO- kidneys were stained for interstitial apoptosis (TUNEL) or proliferation (Ki67) at 3, 7, and 14 days of life and analyzed in 20 high-power fields (hpf) per section at x400. Data are the mean + s.e. (n = 10 in each group).
(TIFF)

S1 Raw Images Westernblot documents. (PDF)
