# Supplementary figures and images for: Tyrphostin AG490 reduces inflammation and fibrosis in neonatal obstructive nephropathy
Source: PLoS One. 2019 Dec 17;14(12):e0226675. doi: 10.1371/journal.pone.0226675 (PMC6917291; doi:10.1371/journal.pone.0226675)

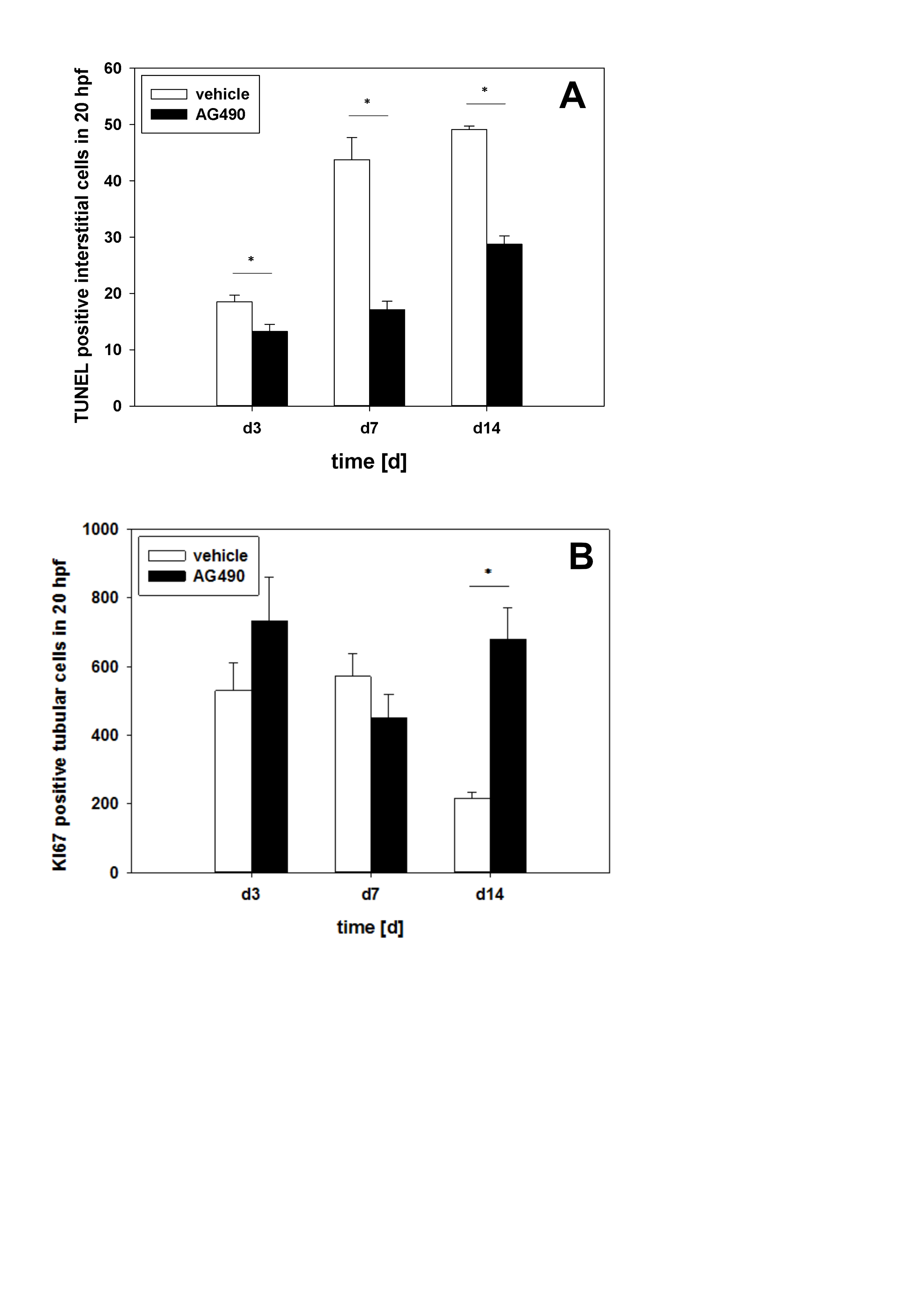

Supplement: S1 Fig — Tyrphostin AG490 reduced interstitial apoptosis (A) and increased tubular proliferation (B) in obstructed kidneys after unilateral ureteral obstruction (UUO) in neonatal mice. Mice received Tyrphostin AG490 or vehicle by daily injections. Renal sections of UUO- kidneys were stained for interstitial apoptosis (TUNEL) or proliferation (Ki67) at 3, 7, and 14 days of life and analyzed in 20 high-power fields (hpf) per section at x400. Data are the mean + s.e. (n = 10 in each group). (TIFF) [file pone.0226675.s002.tiff]
