# Supplementary material for: Tyrphostin AG490 reduces inflammation and fibrosis in neonatal obstructive nephropathy
Source: PLoS One. 2019 Dec 17;14(12):e0226675. doi: 10.1371/journal.pone.0226675 (PMC6917291; doi:10.1371/journal.pone.0226675)
Supplement: S1 Raw Images — (PDF) [file pone.0226675.s003.pdf]

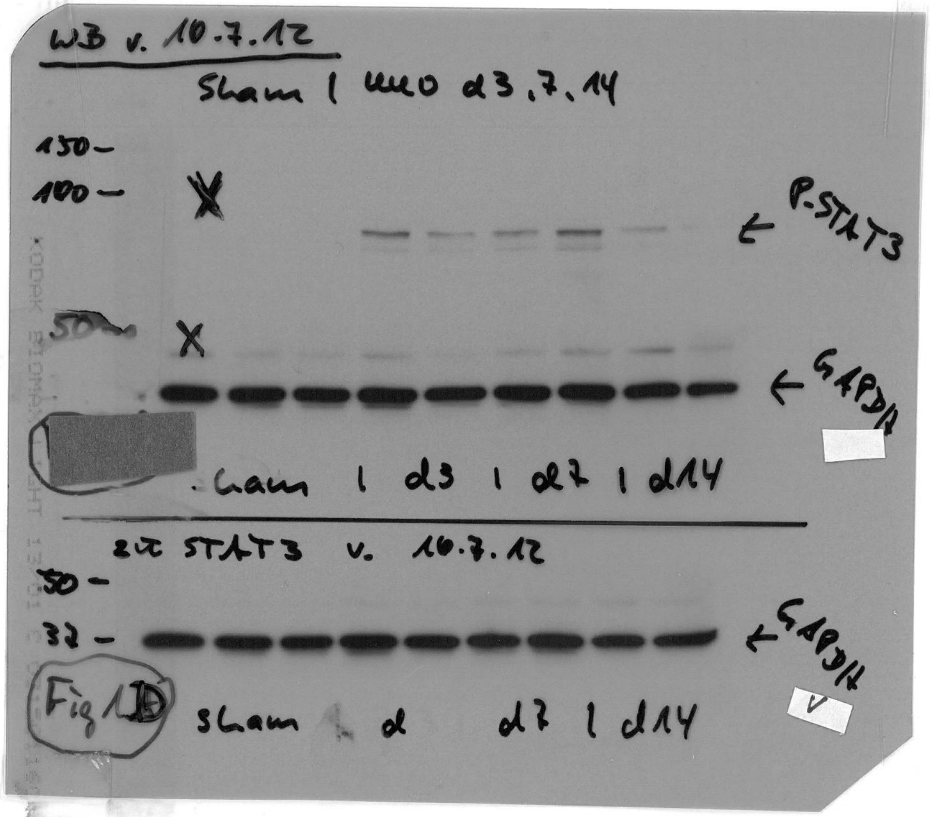
WB v. 10.7.12
Sham | KKO d3, 7, 14
150 –
100 –
P-STAT3
50 –
GAPDH
Sham | d3 | d7 | d14
zu STAT3 v. 10.7.12
50 –
37 –
GAPDH
Fig 1D
Sham d d7 | d14

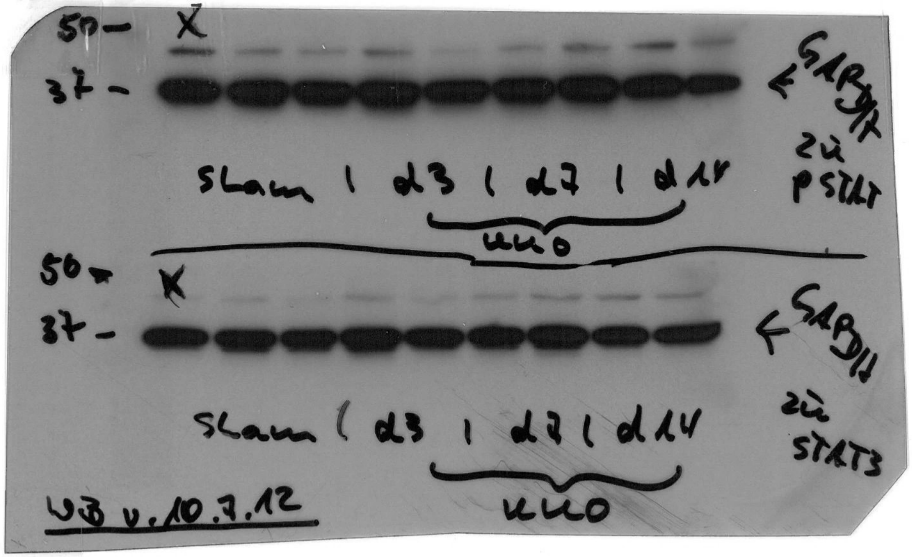
50 –
37 –
GAPDH
zu pSTAT
Sham | d3 | d7 | d14
KKO
50 –
37 –
GAPDH
zu STAT3
Sham | d3 | d7 | d14
KKO
WB v. 10.7.12

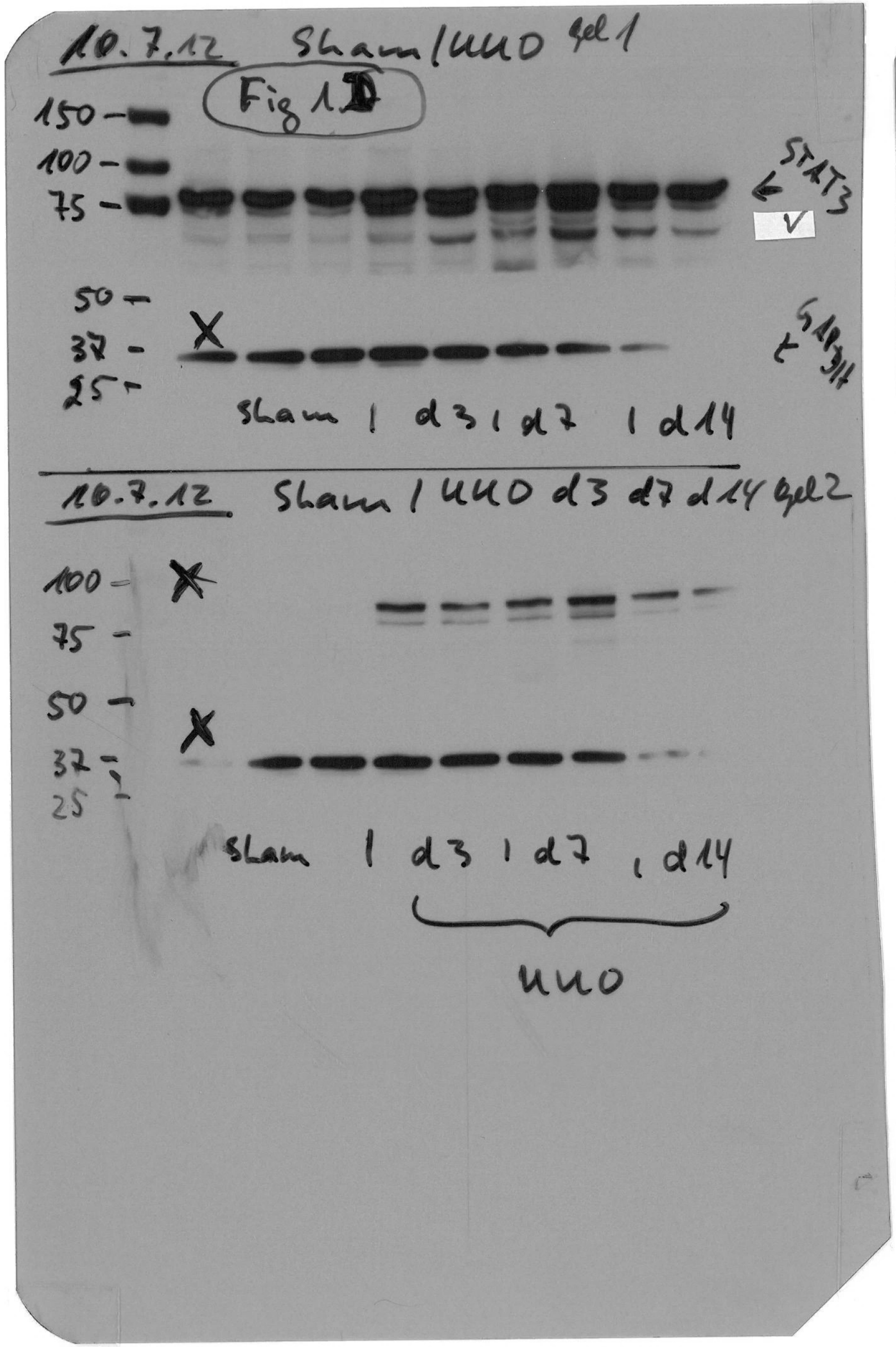

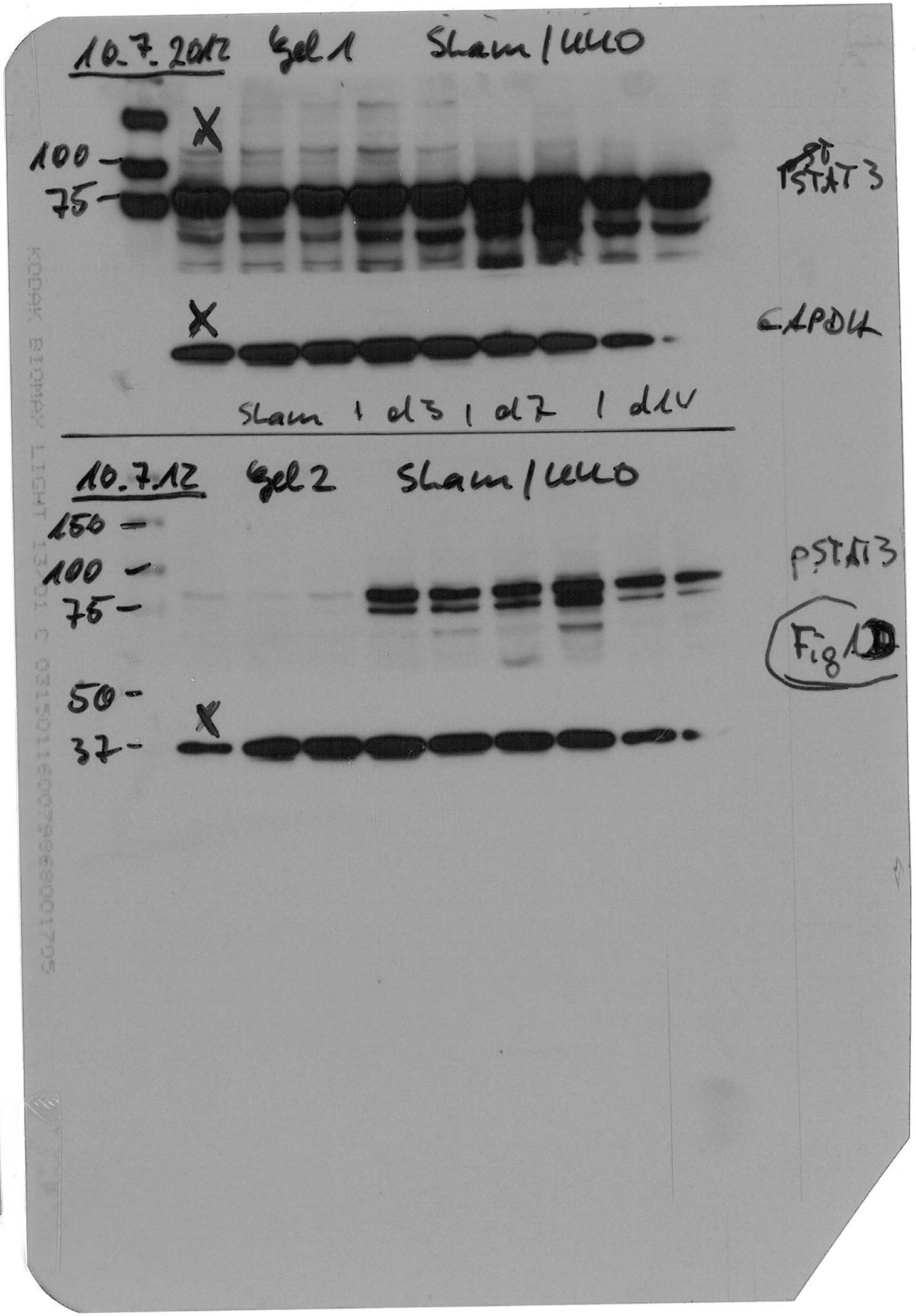

Westernblot v. 10.7.12

STAT-Projekt

pSTAT3

STAT3

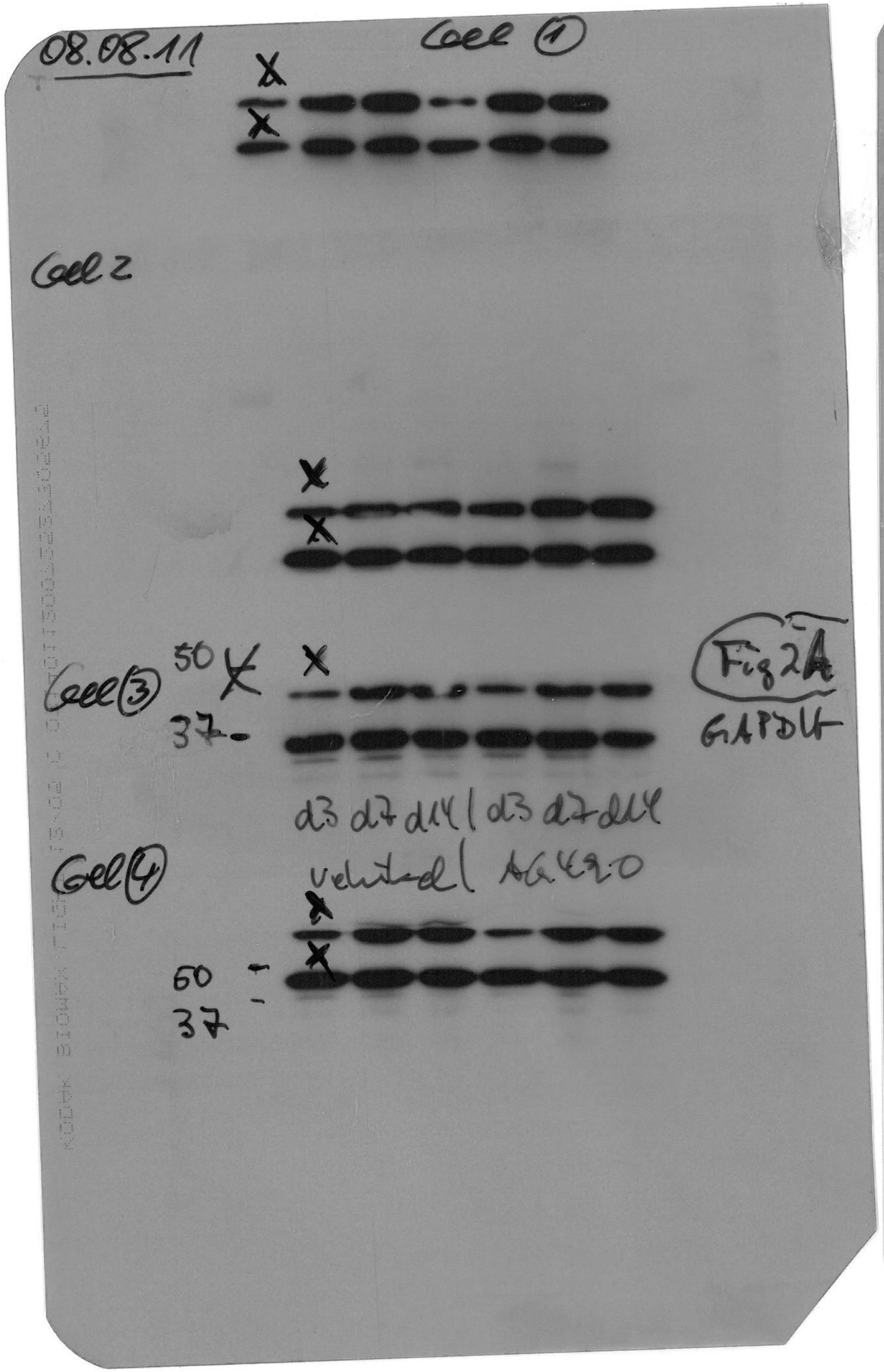
08.08.11
Gel 1
Gel 2
Gel 3
50
37
Fig 2A
GAPDH
d3 d7 d14 | d3 d7 d14
vehicle | AG490
Gel 4
50
37

Gele v. 08.08.11 (uuv)

Nierenlysate (uuo) Vehikel vs. AG490

E-Cadherin, Cyclin B1, D1, PARP, p-STAT3

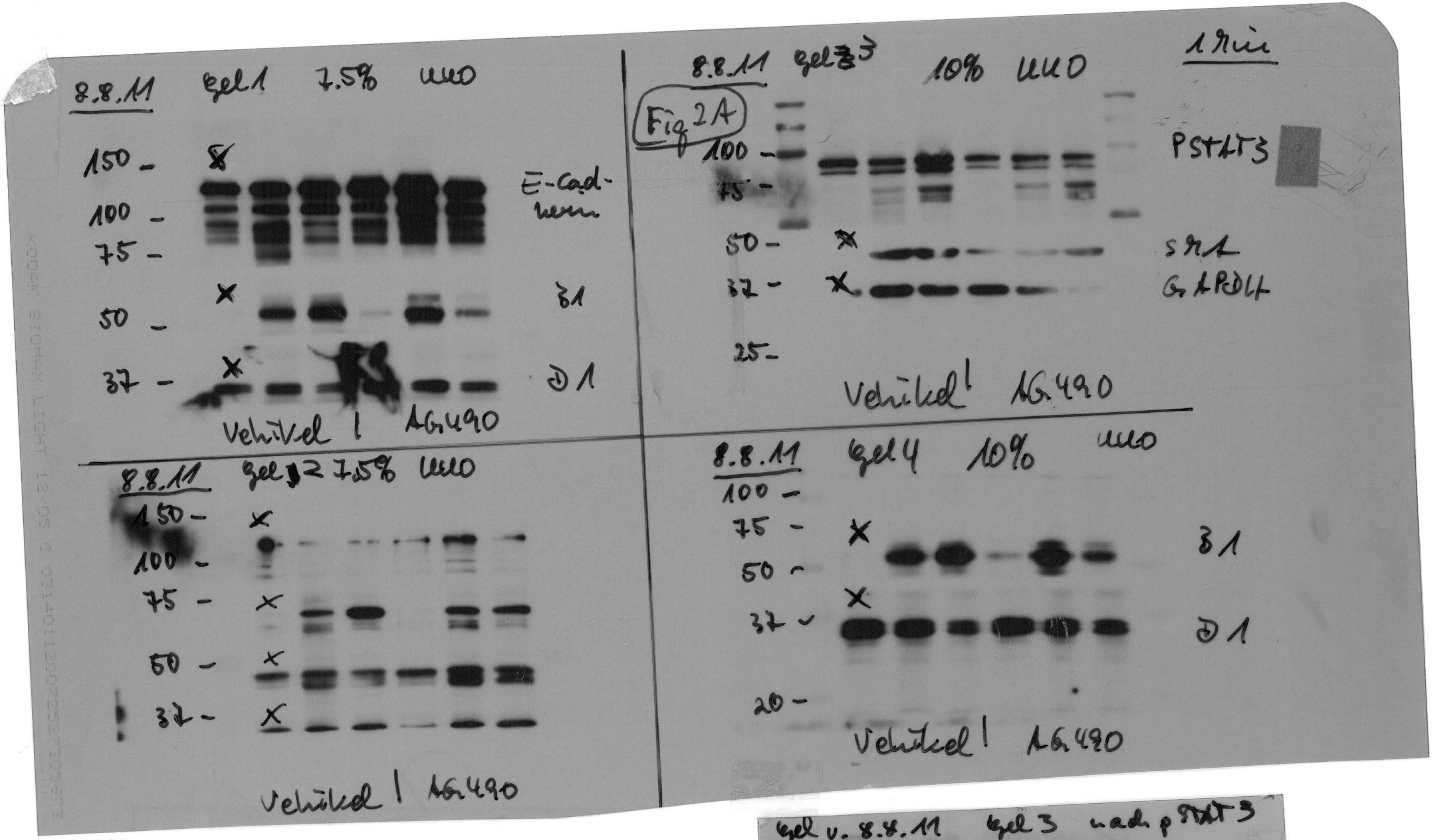

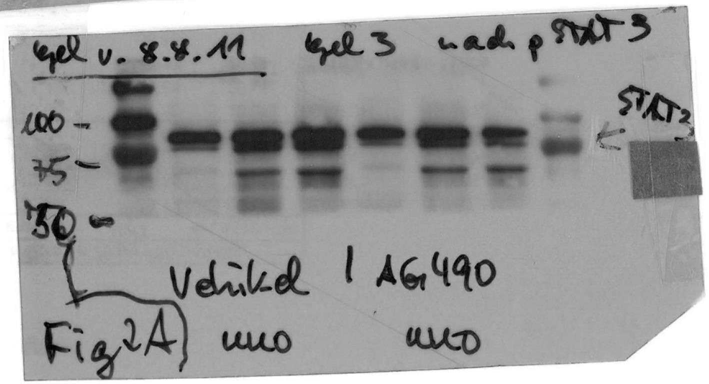

09.08.11

Gele v. 14.09.10 15.09.10

PKSV-PR Lysate (aus HD, 0-20% Stretch)

P-STAT3 + GAPDH

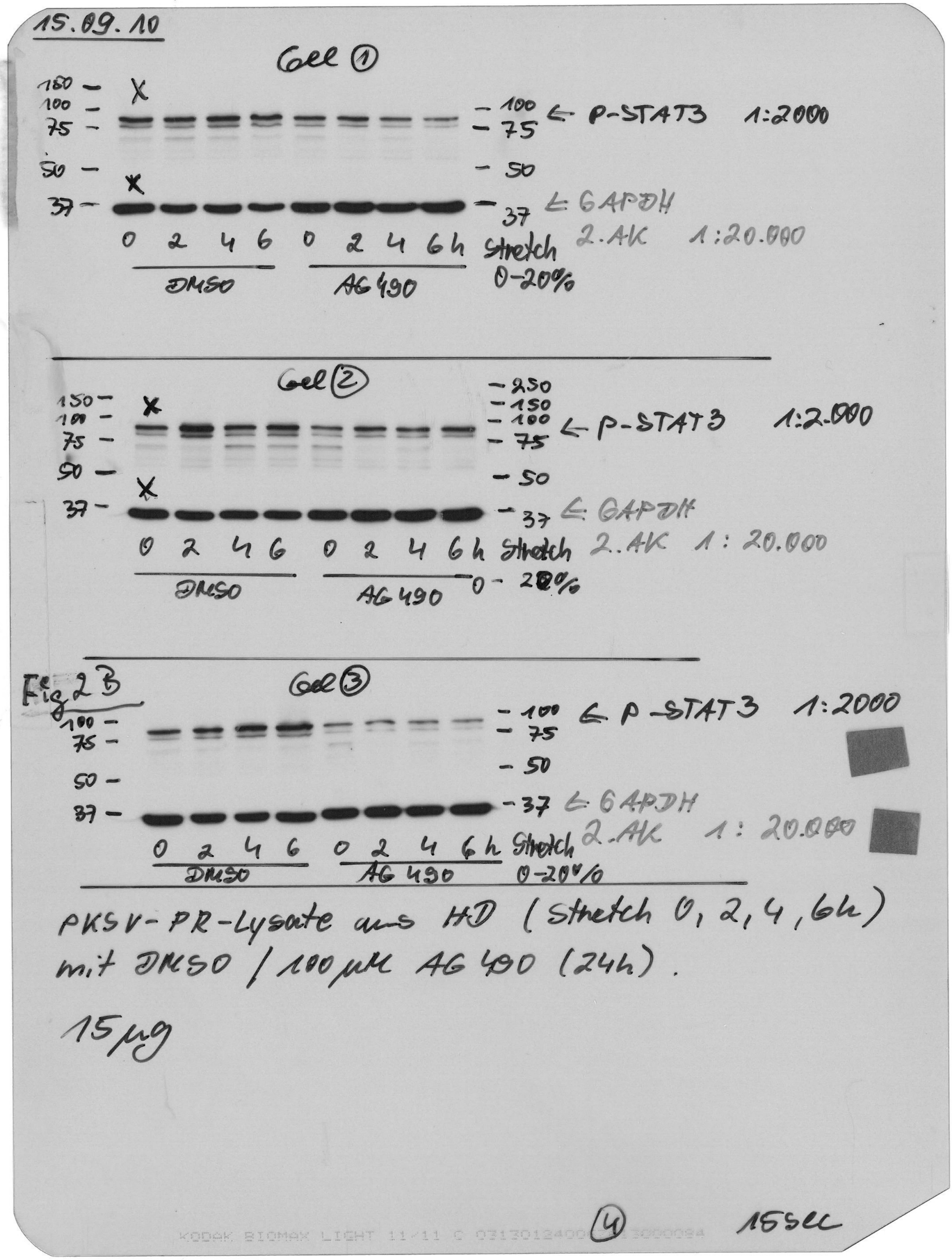

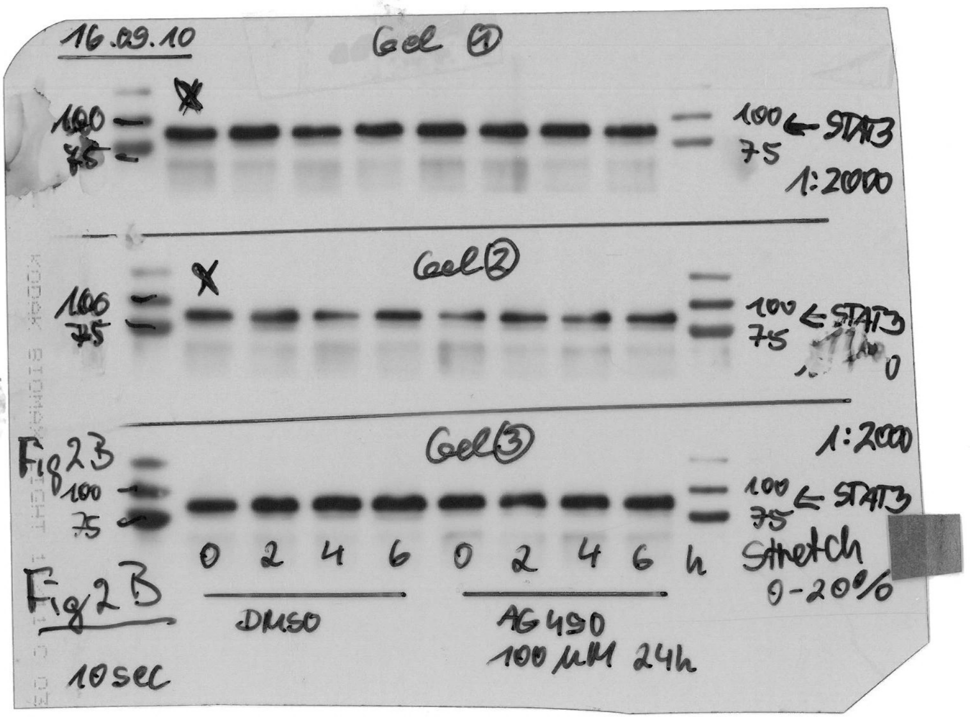
16.09.10
Gel 1
100
75
100 ← STAT3
75
1:2000
Gel 2
100
75
100 ← STAT3
75
Fig 2B
Gel 3
1:2000
100
75
100 ← STAT3
75
0 2 4 6 0 2 4 6 h
Stretch
0-20%
Fig 2B
DMSO
AG490
100 µM 24h
10 sec
KODAK BIOMAX LIGHT

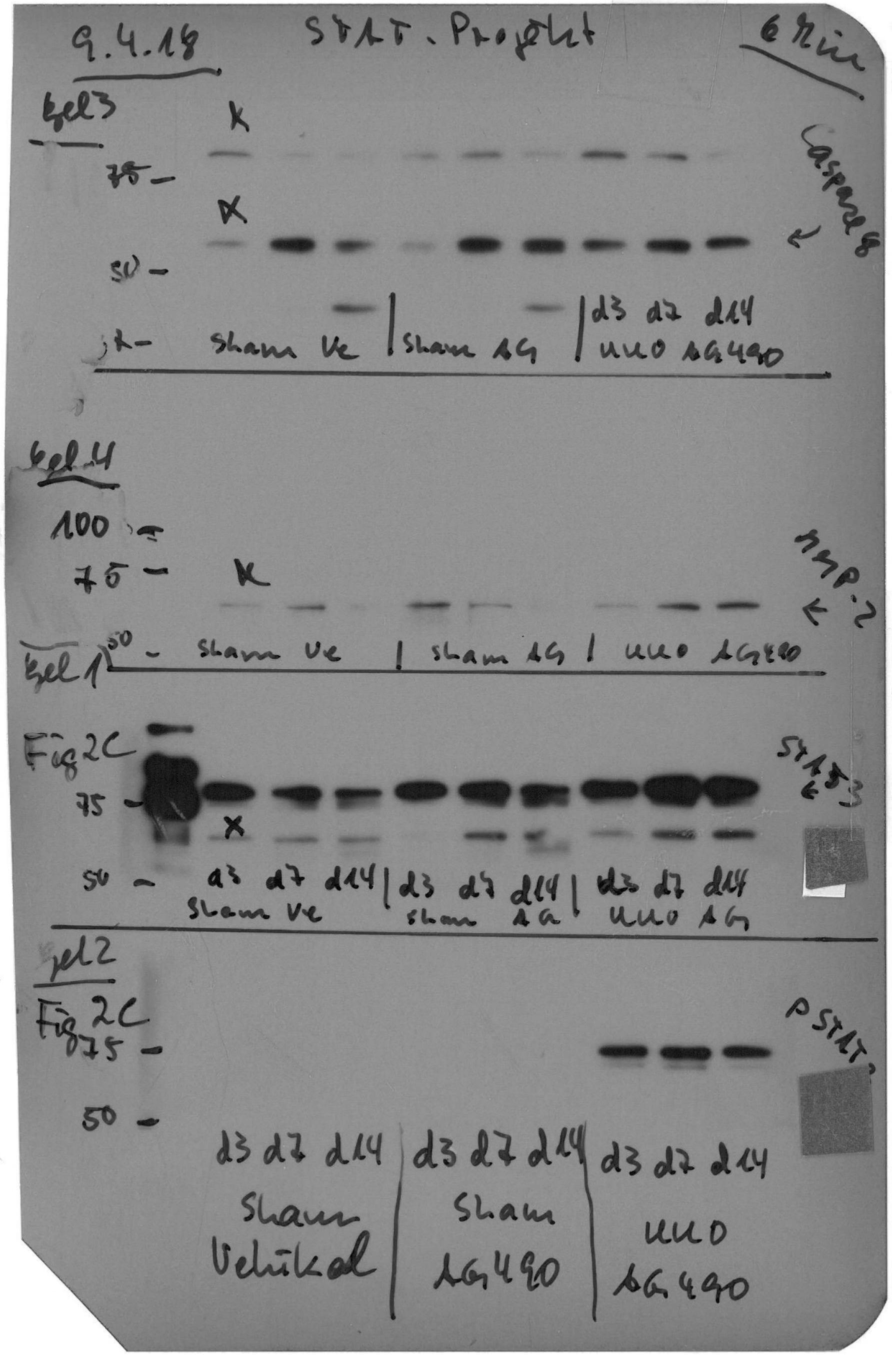
9.4.18
STAT-Projekt
Gel3
75 –
50 –
Sham Ve
Sham AG
d3 d7 d14
UUO AG490
Gel4
100 –
75 –
50 –
Sham Ve
Sham AG
UUO AG490
Gel1
Fig2C
75 –
50 –
d3 d7 d14
Sham Ve
d3 d7 d14
Sham AG
d3 d7 d14
UUO AG
Gel2
Fig2C
75 –
50 –
d3 d7 d14
Sham
Vehikel
d3 d7 d14
Sham
AG490
d3 d7 d14
UUO
AG490

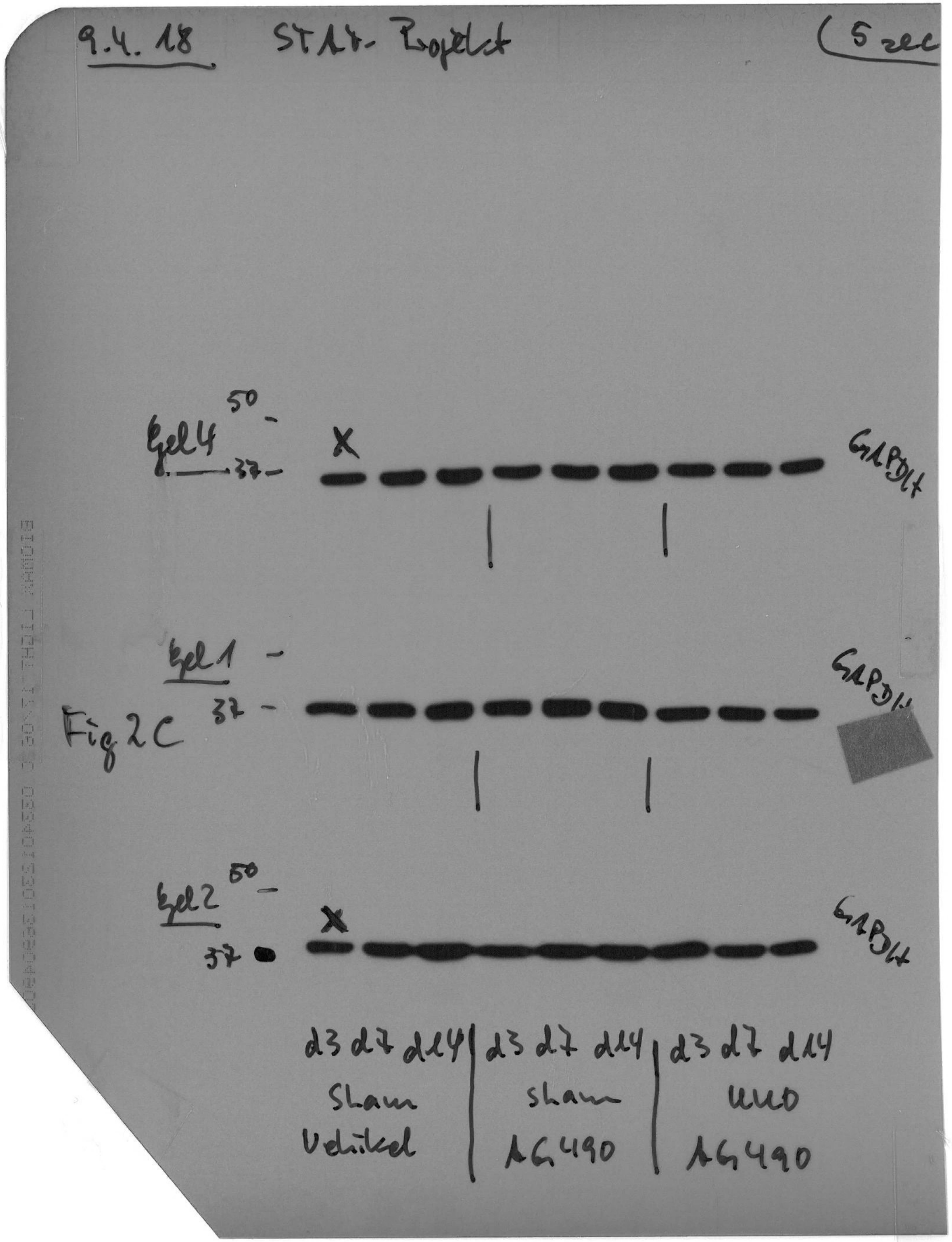
9.4.18
STAT-Projekt
(5 sec
Gel4
50 –
37 –
GAPDH
Gel1
Fig2C
37 –
GAPDH
Gel2
50 –
37
GAPDH
d3 d7 d14
Sham
Vehikel
d3 d7 d14
Sham
AG490
d3 d7 d14
UUO
AG490

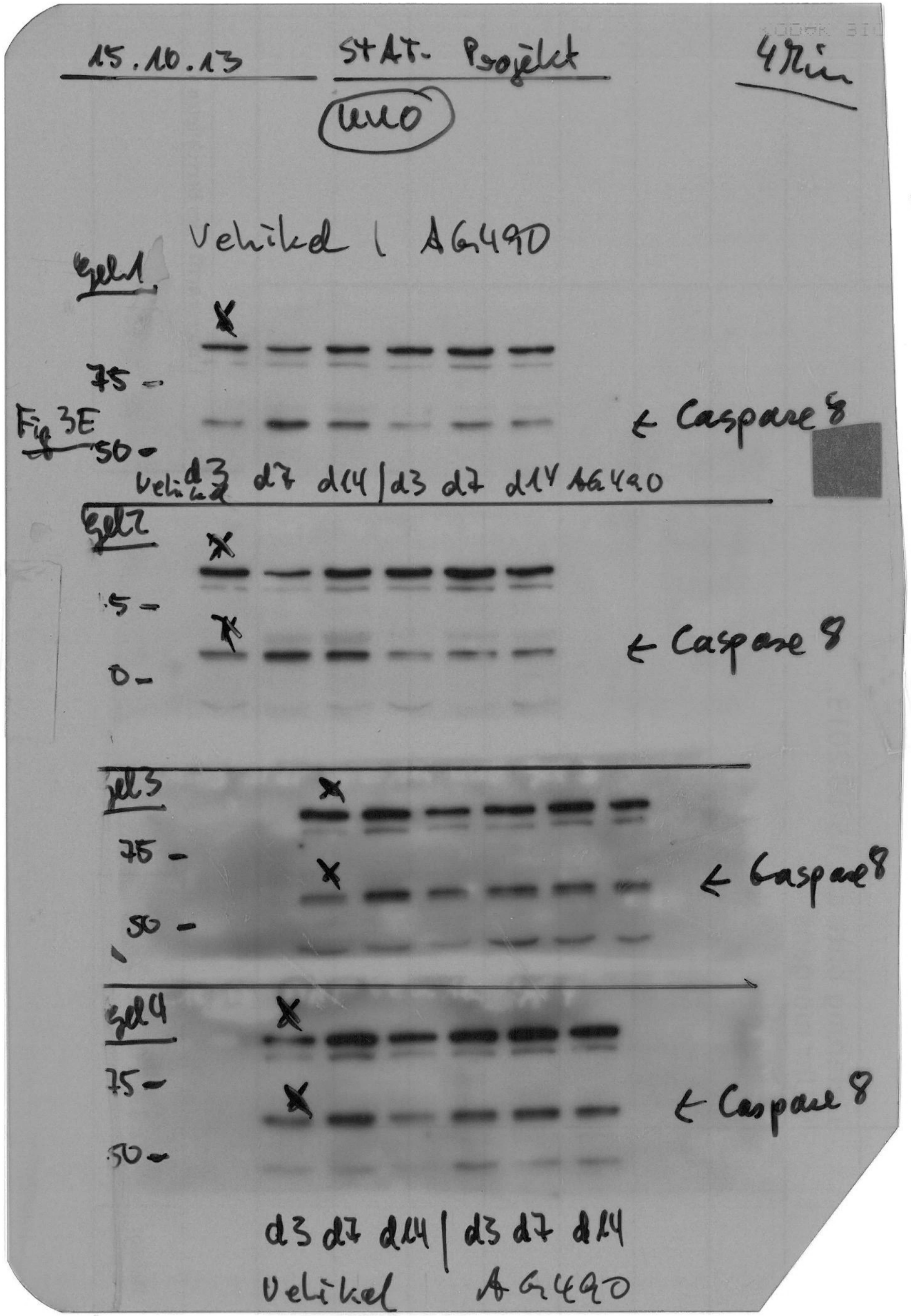

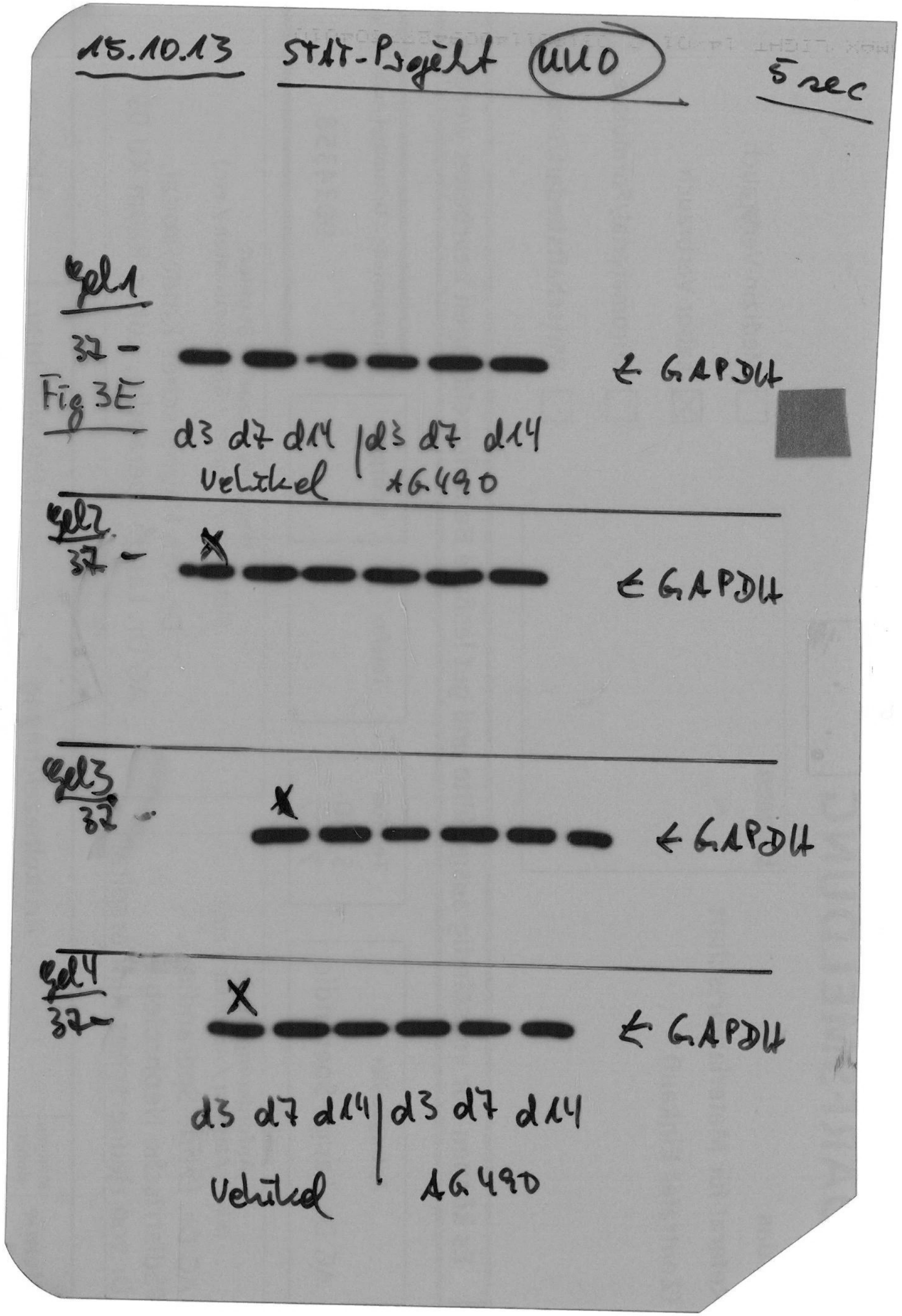

15.10.13 Westernblot STAT-Projekt Caspase 8 GAPDH

Standard Curve(s)

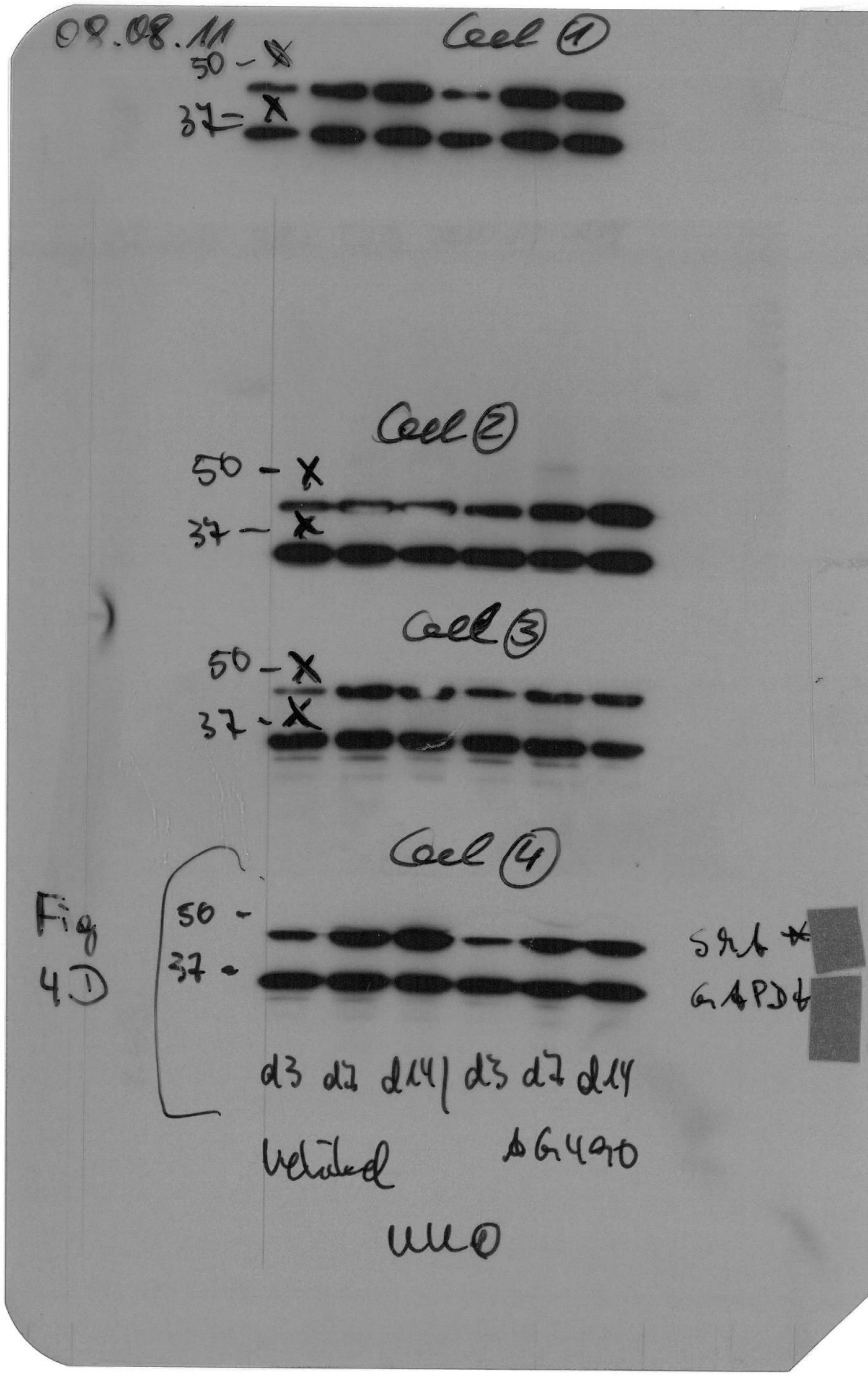

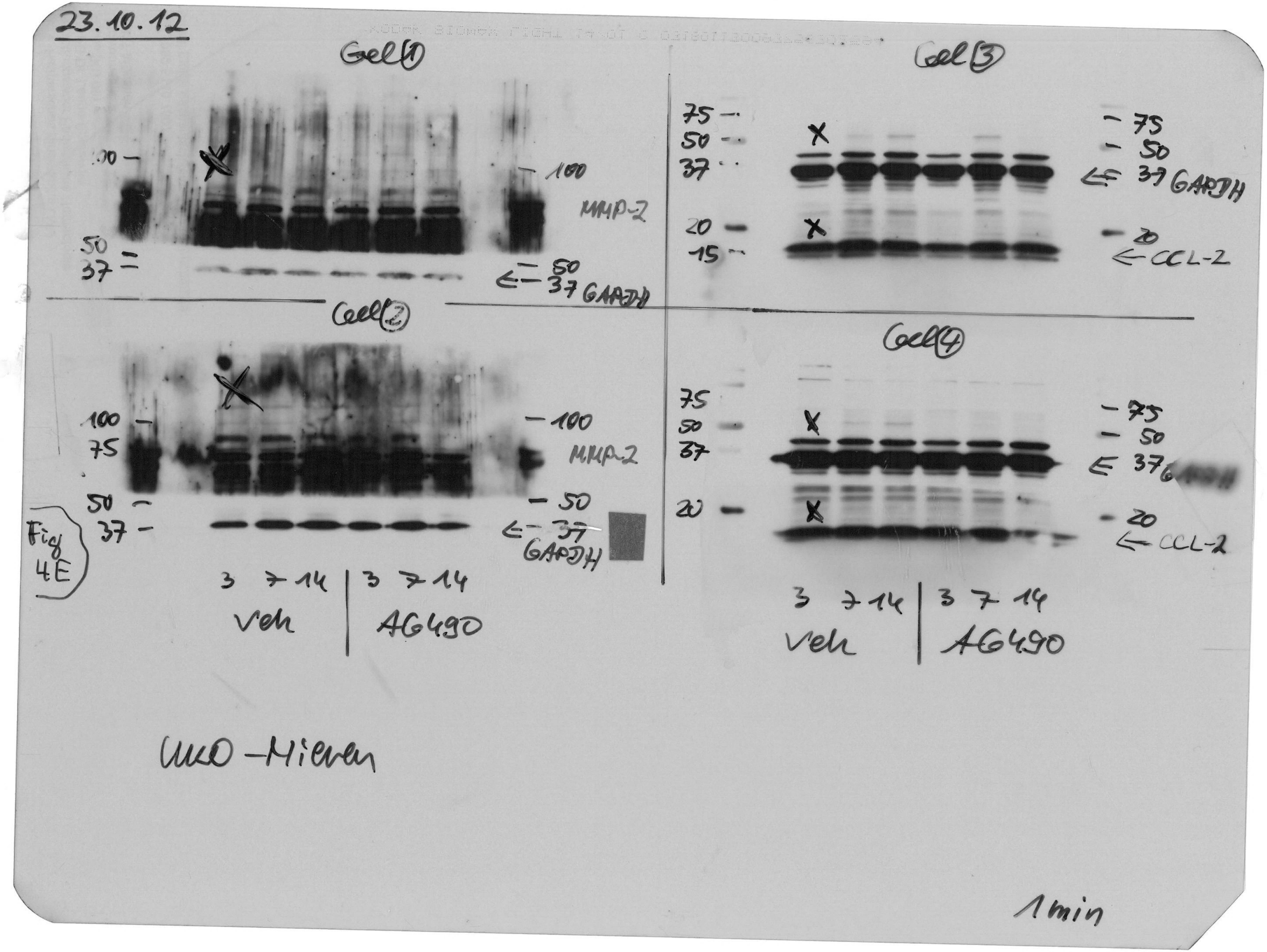
23.10.12
Gel 1
100
100
MMP-2
50
37
50
37 GAPDH
Gel 3
75
50
37
20
15
75
50
37 GAPDH
20
CCL-2
Gel 2
100
75
100
MMP-2
50
37
50
37
GAPDH
Fig 4E
3 7 14 | 3 7 14
veh | AG490
Gel 4
75
50
37
20
75
50
37
20
CCL-2
3 7 14 | 3 7 14
veh | AG490
UkO-Nieren
1min

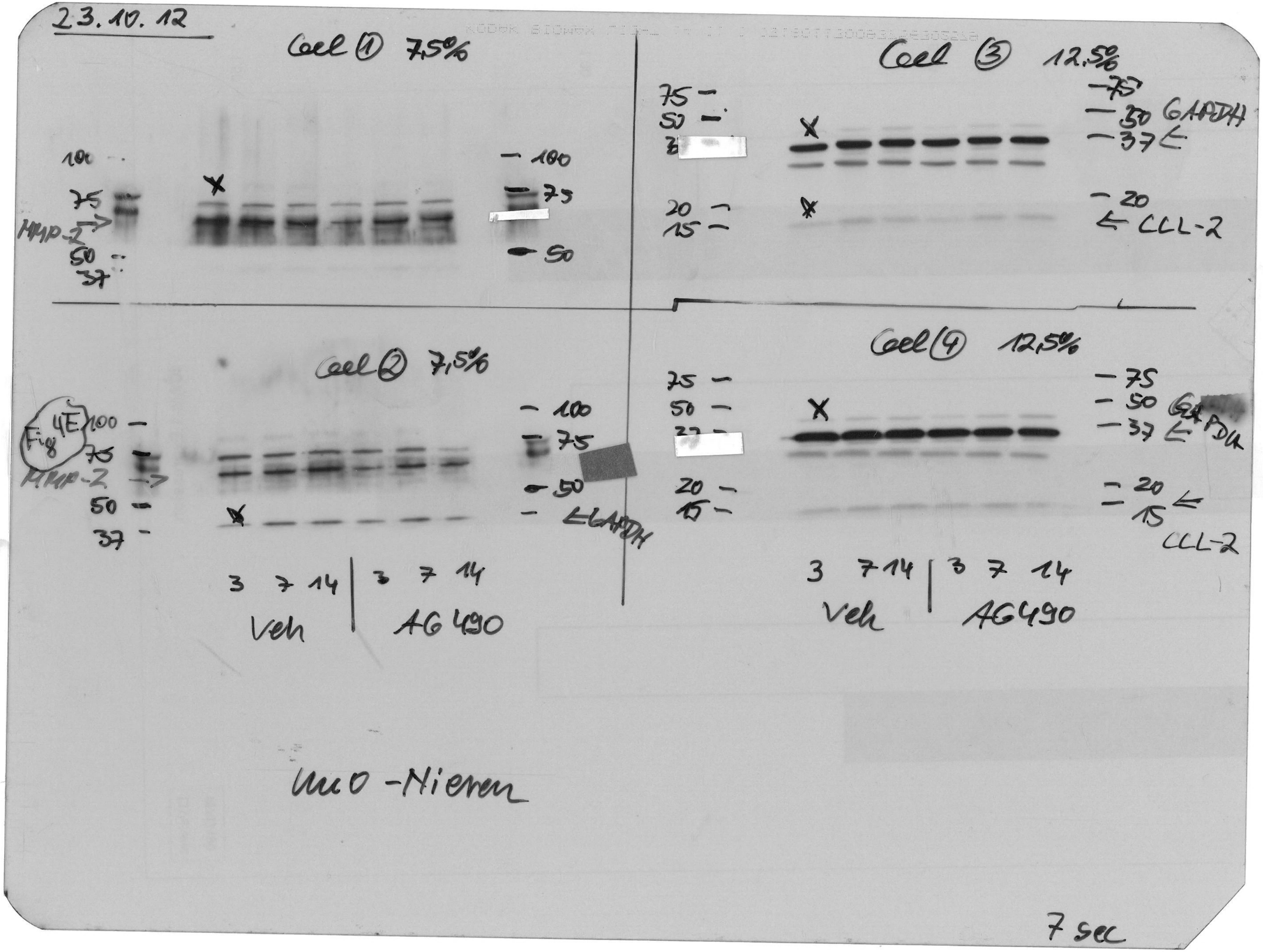
23.10.12
Gel ① 7,5%
Gel ③ 12,5%
MMP-2
GAPDH
CCL-2
Gel ② 7,5%
Gel ④ 12,5%
Fig 4E
Fig 8
3 7 14 | 3 7 14
Veh
AG490
UUO-Nieren
7 sec

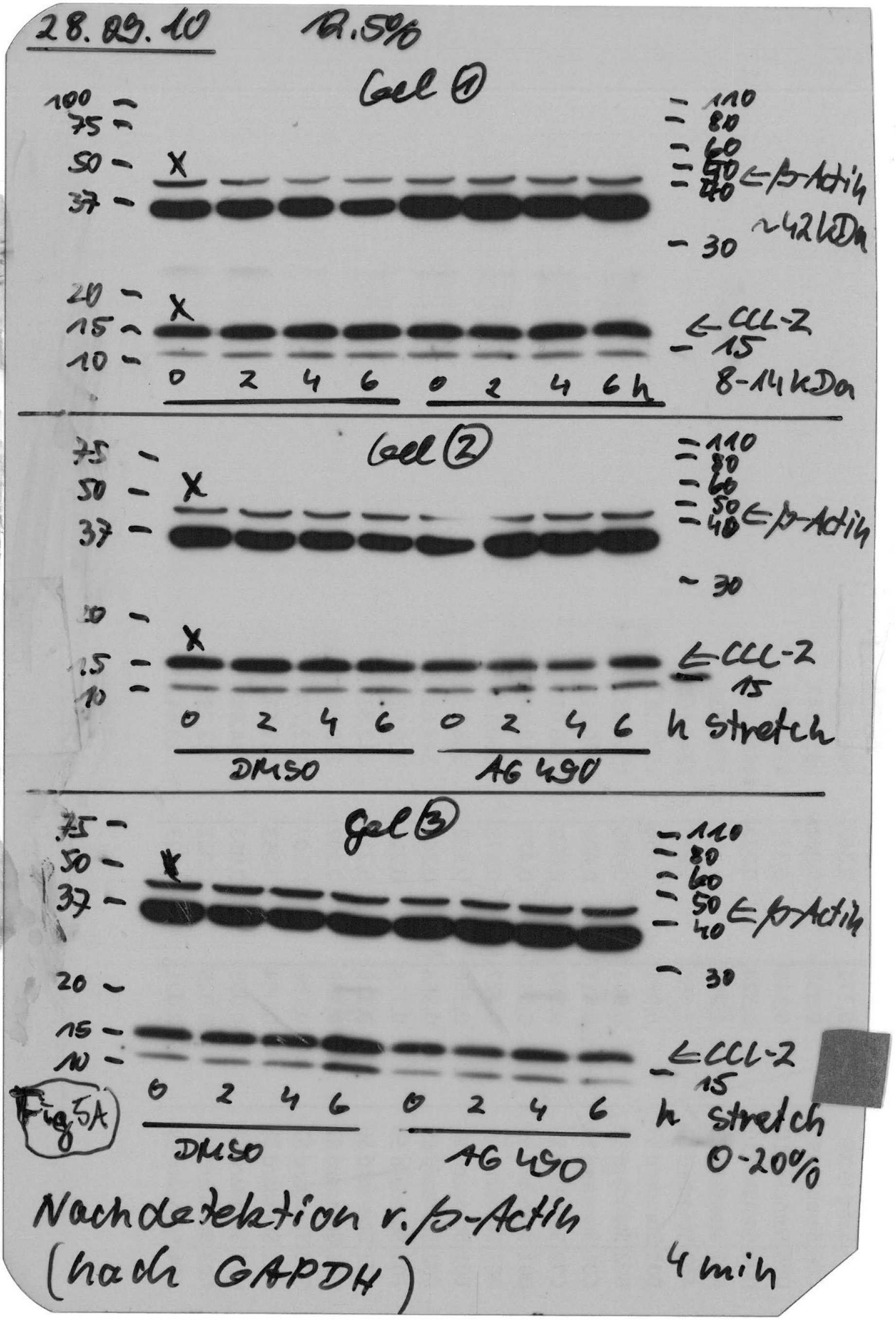
28.09.10 12.5%
Gel ①
100 75 50 37
20 15 10
110 80 60 50 40 30
← β-Actin ~42 kDa
← CCL-2 15
0 2 4 6 0 2 4 6 h 8-14 kDa
Gel ②
75 50 37
20 15 10
110 80 60 50 40 30
← β-Actin
← CCL-2 15
0 2 4 6 0 2 4 6 h stretch
DMSO AG 490
Gel ③
75 50 37
20 15 10
110 80 60 50 40 30
← β-Actin
← CCL-2 15
0 2 4 6 0 2 4 6 h stretch 0-20%
DMSO AG 490
Fig 5A
Nachdetektion v. β-Actin
(nach GAPDH)
4 min

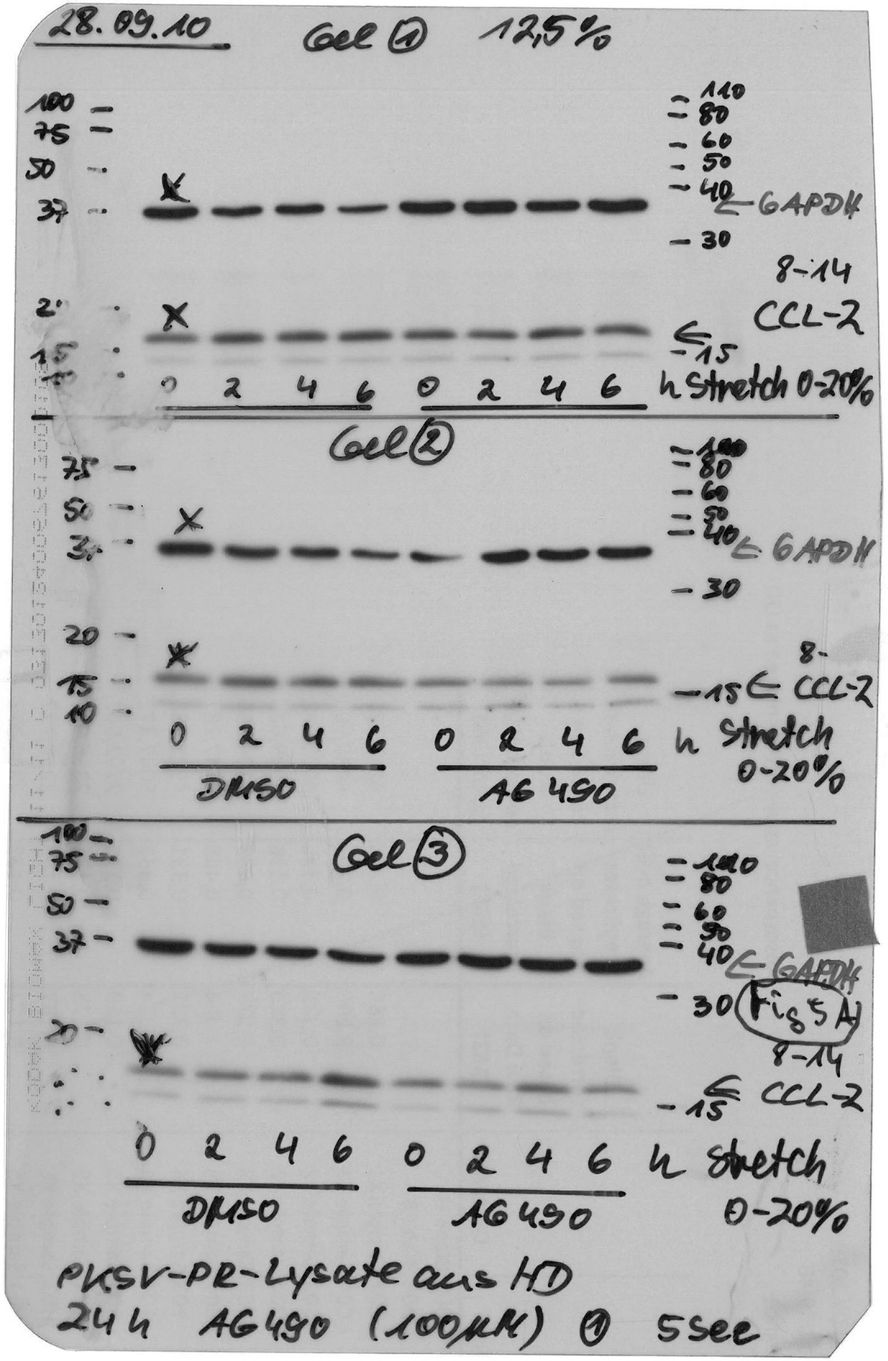
28.09.10 Gel ① 12,5%
100 75 50 37
15
110 80 60 50 40 30
← GAPDH
8-14 CCL-2
← 15
0 2 4 6 0 2 4 6 h stretch 0-20%
Gel ②
75 50 37
20 15 10
100 80 60 50 40 30
← GAPDH
8- ← CCL-2 15
0 2 4 6 0 2 4 6 h stretch 0-20%
DMSO AG 490
Gel ③
100 75 50 37
20
110 80 60 50 40 30
← GAPDH
Fig 5A
8-14 ← CCL-2 15
0 2 4 6 0 2 4 6 h stretch 0-20%
DMSO AG 490
PKSV-PR-Lysate aus HD
24 h AG490 (100 µM) ① 5 sec

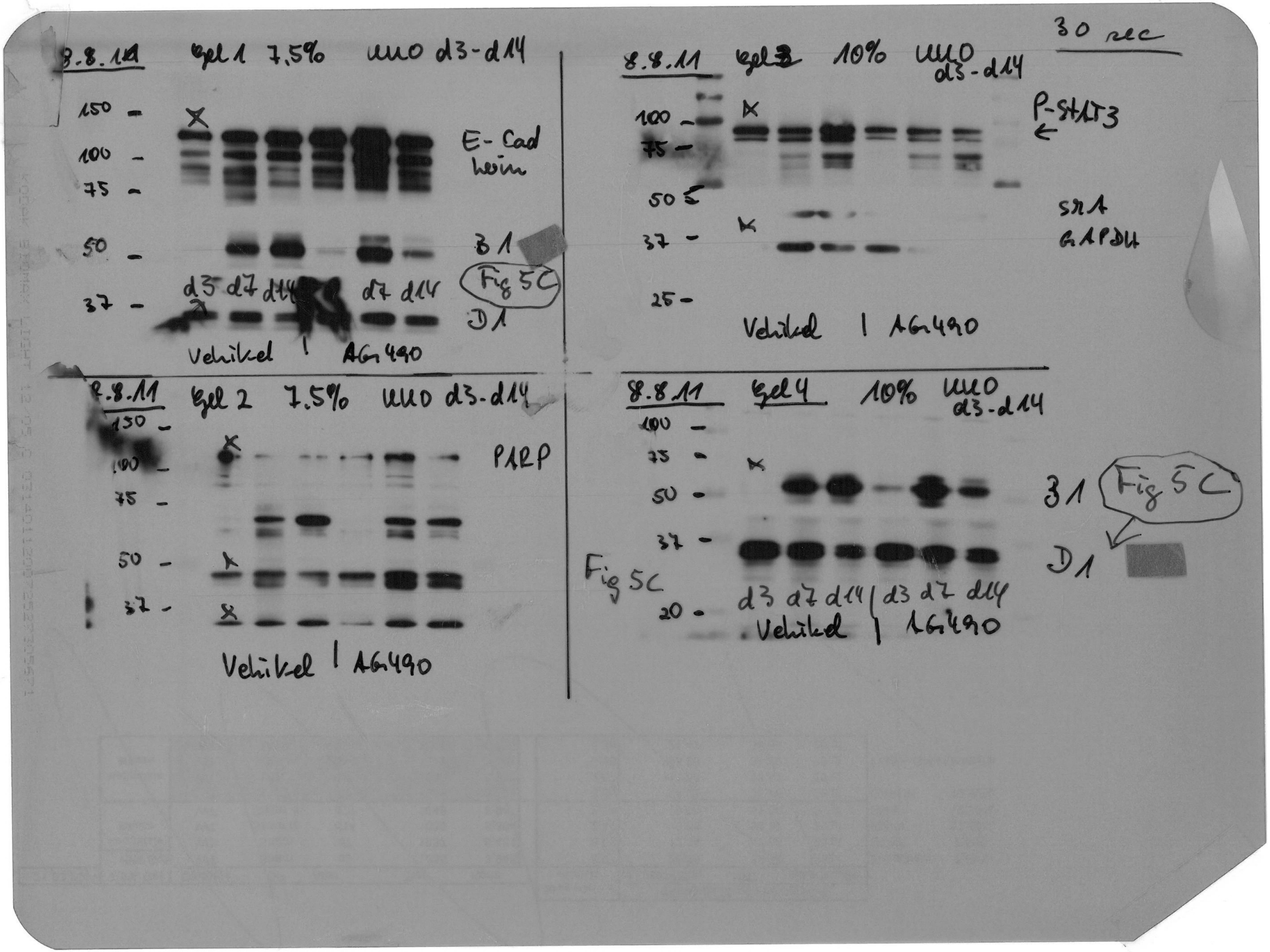

Gele v. 08.08.11 (ULK)

Nieren Lysate (UUO) Vehikel vs AG490 (neo)

E-Cadherin, Cyclin B1, D1, PARP, P-STAT3

08.08.11

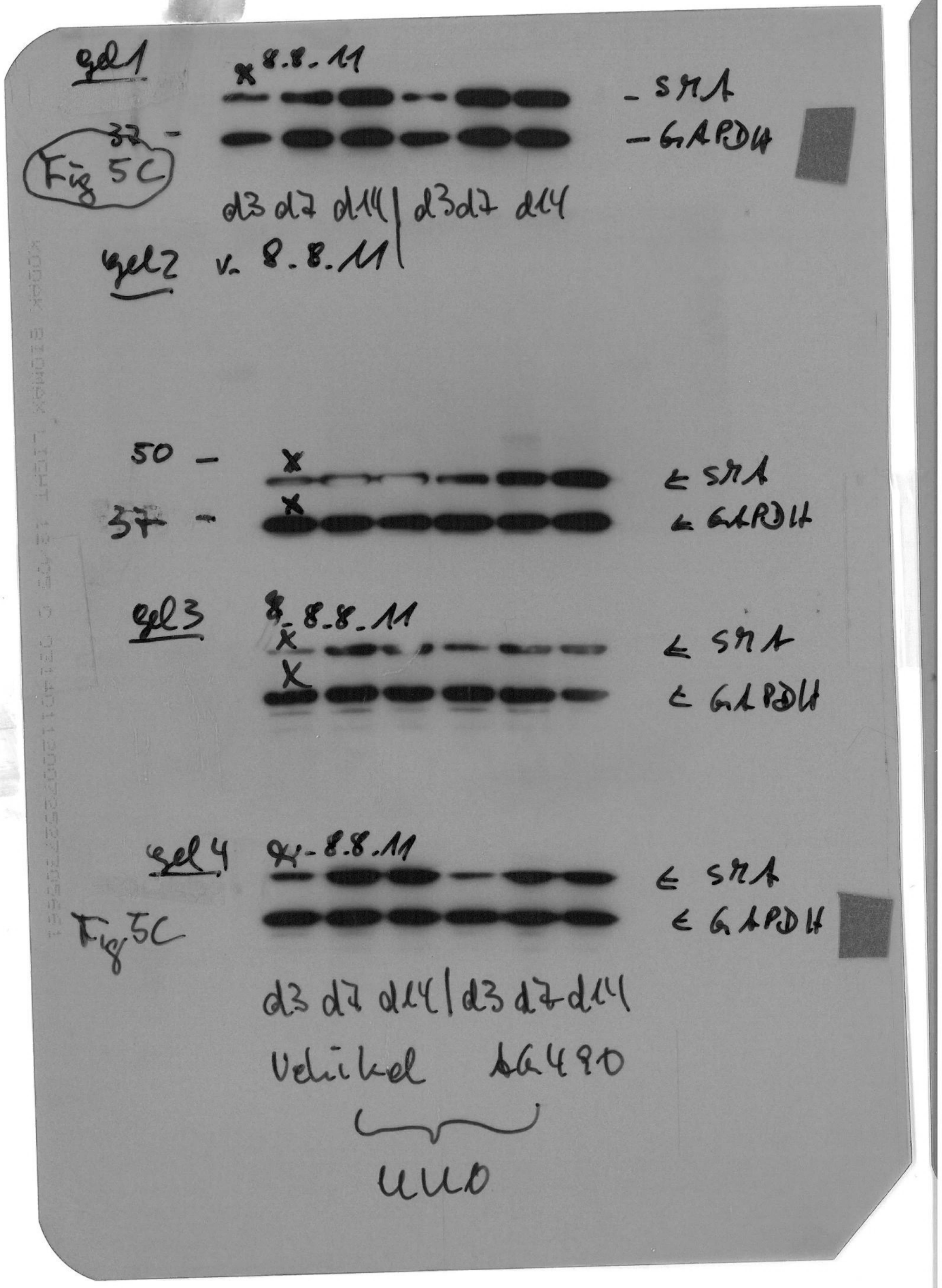
gel1
8.8.11
37 -
Fig 5C
- SMA
- GAPDH
d3 d7 d14 | d3 d7 d14
gel2 v. 8.8.11
50 -
37 -
← SMA
← GAPDH
gel3
8.8.11
← SMA
← GAPDH
gel4
8.8.11
← SMA
← GAPDH
Fig 5C
d3 d7 d14 | d3 d7 d14
Vehikel AG490
UUO

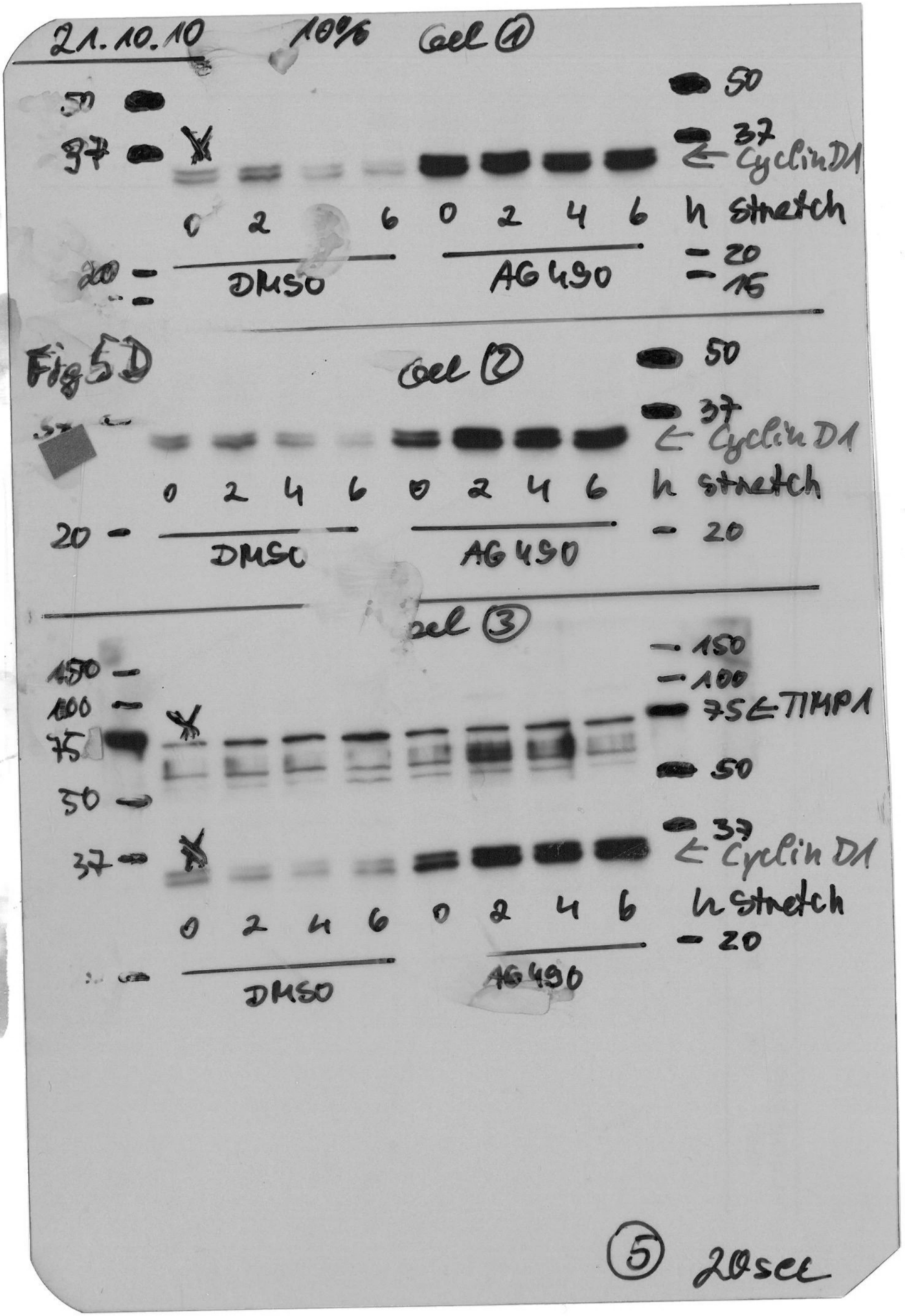
21.10.10
10%
Gel 1
50
37
Cyclin D1
0 2 6 0 2 4 6 h stretch
20
15
DMSO
AG490
Fig 5D
Gel 2
Cyclin D1
0 2 4 6 0 2 4 6 h stretch
DMSO
AG490
Gel 3
150
100
75
TIMP1
Cyclin D1
0 2 4 6 0 2 4 6 h stretch
DMSO
AG490
5
20sec

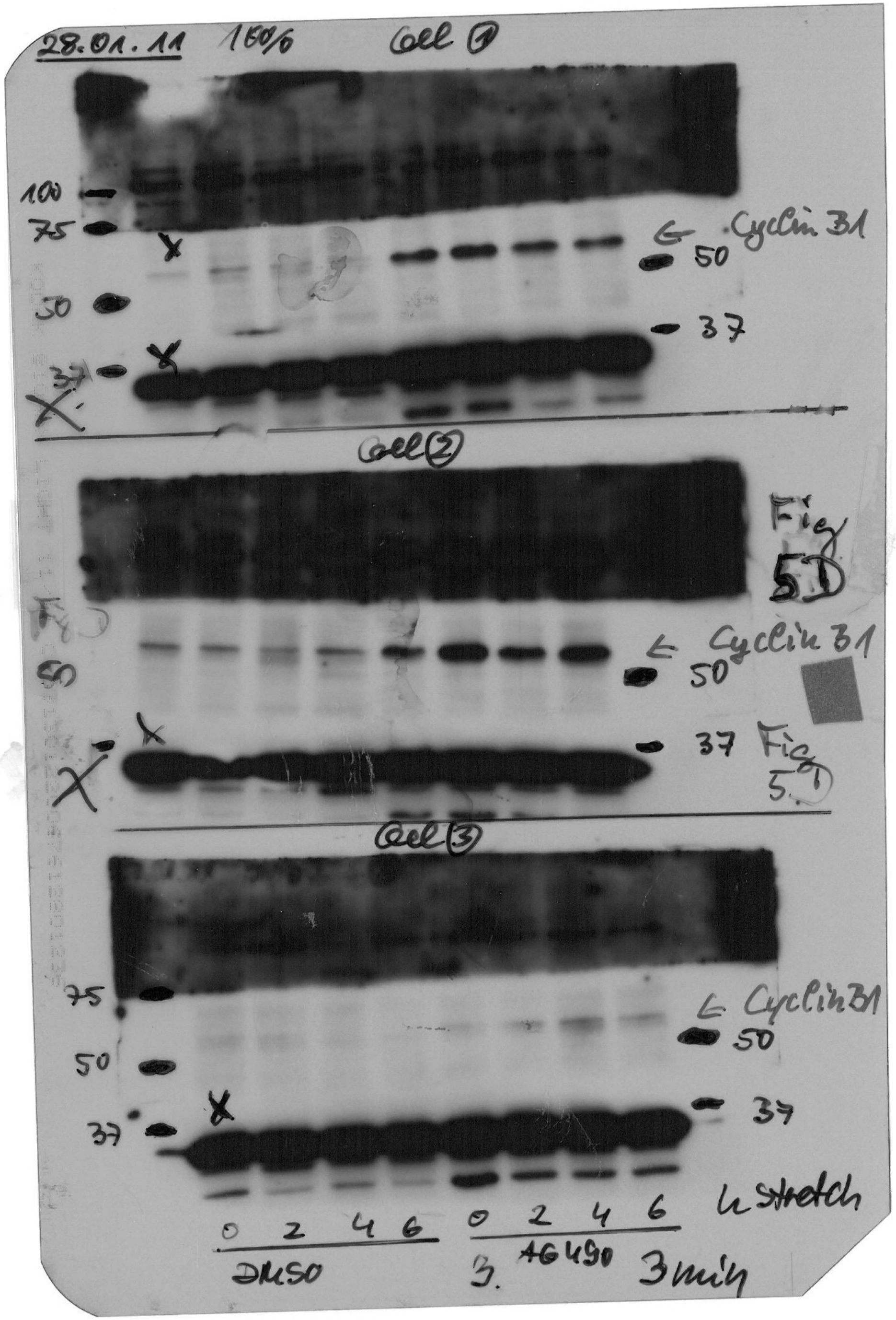
28.01.11
10%
Gel 1
100
75
Cyclin B1
50
37
Gel 2
Fig 5D
Cyclin B1
Fig 5.D
Gel 3
Cyclin B1
h stretch
0 2 4 6 0 2 4 6
DMSO
AG490
3.
3min

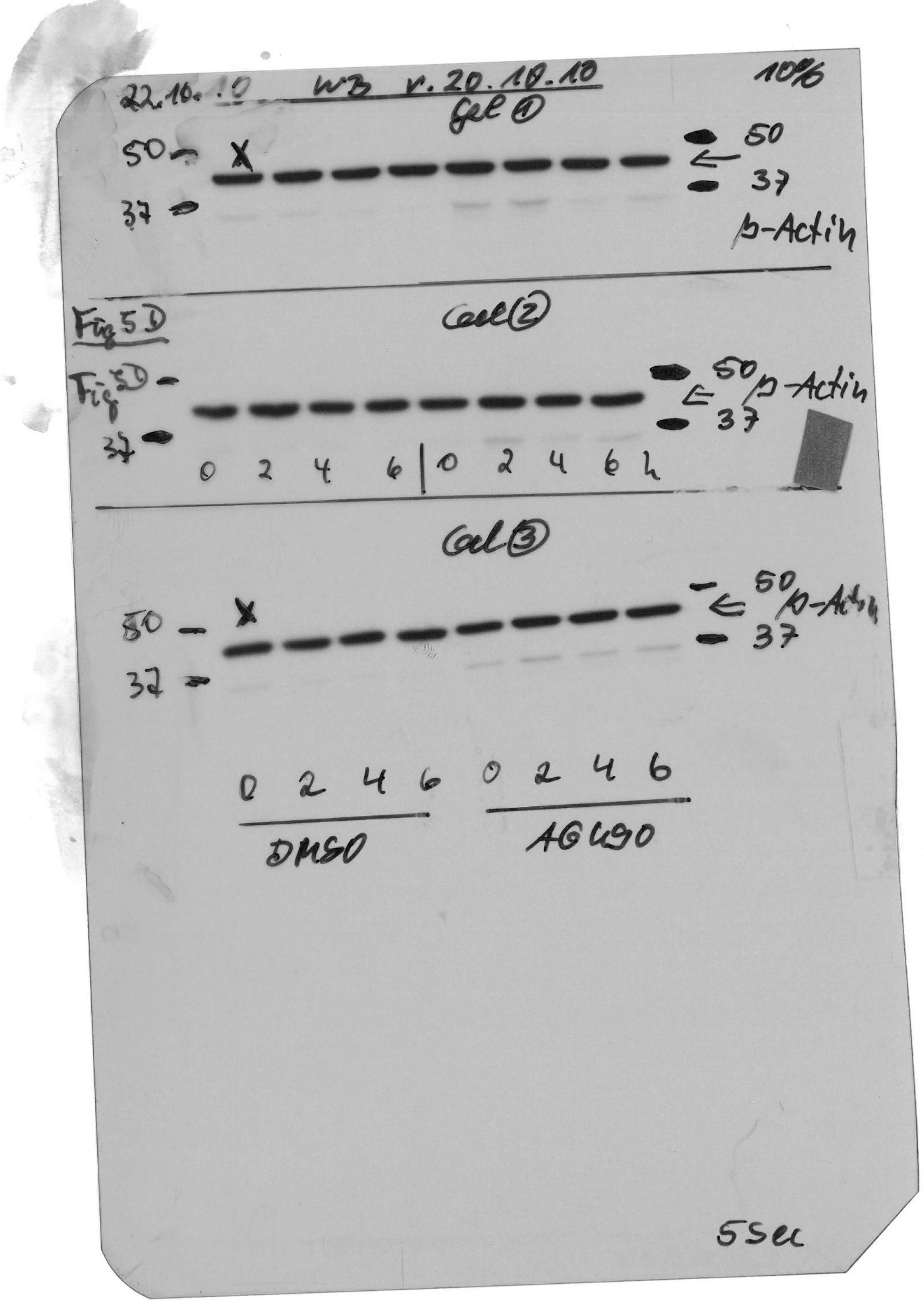
22.10.10 WB v. 20.10.10
10%
Gel ①
50
37
50
37
β-Actin
Fig 5D
Gel ②
50
37
50
37
β-Actin
0 2 4 6 | 0 2 4 6 h
Gel ③
50
37
50
37
β-Actin
0 2 4 6 0 2 4 6
DMSO
AG490
5sec
